# Planetary shielding and surface precipitation of solar energetic particles during BepiColombo’s close Mercury flyby

**DOI:** 10.1038/s41550-026-02914-6

**Published:** 2026-09-08

**Authors:** Emilia K. J. Kilpua, Rami Vainio, Manuel Grande, Liam Edwards, Monica Laurenza, Eero Esko, Arto Lehtolainen, Christian Palmroos, Jan Gieseler, Philipp Oleynik, George Ho, David J. Lawrence, Scige John Liu, Stefano Massetti, Daniel Heyner, Kristin Pump, Beatriz Sanchez-Cano, Juhani Huovelin

**Affiliations:** 1 Department of Physics, University of Helsinki, Helsinki, Finland; 2 Department of Physics and Astronomy, University of Turku, Turku, Finland; 3 Department of Physics, Aberystwyth University, Cymru, UK; 4 INAF-Istituto di Astrofisica e Planetologia Spaziali, Rome, Italy; 5 Southwest Research Institute, San Antonio, TX USA; 6 Johns Hopkins University Applied Physics Laboratory, Laurel, MD USA; 7 Technische Universität Braunschweig, Institute for Geophysics and extraterrestrial Physics, Braunschweig, Germany; 8 University of Leicester, Leicester, UK; 9 Isaware Oy, Helsinki, Finland

**Keywords:** Inner planets, Inner planets

## Abstract

Planetary magnetic fields act as protective barriers, shielding a planet’s atmosphere and surface from energetic charged particles originating from its parent star and outer space. During its fourth flyby of Mercury on 4 September 2024, the ESA/JAXA BepiColombo mission passed within just 165 km of the planet’s surface. This close approach coincided with the elevated fluxes of high-energy charged solar particles, providing an opportunity to study shielding processes of a planet in close proximity to its star. Here, utilizing observations from the Solar Intensity X-Ray and Particle Spectrometer (SIXS) onboard BepiColombo, we investigate the penetration of high energy particles from the Sun into Mercury’s magnetosphere. The variations in energetic particle fluxes depend on the particle type, direction and energy. Our analysis reveals that the disappearance of protons is related to magnetic shadowing by the planet, while electrons dropouts occur due to a wide magnetic loss cone. These findings show that sustained high-energy particle precipitation can occur during solar energetic particle events on weakly magnetized rocky planets orbiting close to their parent star. Such processes probably play a significant role in regolith alteration and exospheric variability, with broader implications for surface geochemistry and potential habitability.

## Abstract (web summary)

BepiColombo’s flyby at just 165 km from Mercury observed sustained precipitation of energetic solar protons and electrons onto the planet’s surface, with potentially long-term impact on its weathering and mineralogy.

## Main

Due to its proximity to the Sun, Mercury is strongly influenced by solar storms and solar energetic particles (SEPs)^1–3^. The acceleration of SEPs begins in the low corona, driven by solar flares and coronal mass ejections (CMEs)—huge eruptions of magnetized plasma from the Sun. This process continues in interplanetary space, where shock waves, driven by fast CMEs propagating up to several thousands of kilometres per second, further energize these particles^4,5^.

The ability of SEPs to penetrate a planet’s magnetic shielding depends both on their energy and the field’s strength. Mercury’s weak, off-centre magnetic field^6,7^, about 1% as strong as the Earth’s, results in a relatively small magnetosphere with its sunward boundary located only about 3,570 km (~1.47 Mercury’s radii) from the surface^8,9^. The planet lacks a significant atmosphere and is surrounded only by an extremely tenuous exosphere. Consequently, SEPs with sufficiently large gyroradii can directly reach the planet’s surface. The gyroradius of a relativistic particle is defined by *r*_*g*_ = *γmv*_⊥_/∣*q*∣*B*, where *γ* is the Lorentz factor, *m* the mass of the particle, *v*_⊥_ the velocity of the particle perpendicular to the magnetic field, *B* the magnetic field strength and *q* the particle’s charge. Alternatively, SEPs may reach the surface by propagating along open magnetic flux tubes connecting the interplanetary magnetic field and the planet, provided that they avoid magnetic mirroring^10,11^. Energetic charged particles striking the surface can trigger various processes, such as releasing neutral atoms such as sodium, potassium and calcium, as well as volatiles, and inducing X-ray fluorescence^12–14^. These processes uniquely link Mercury’s regolith, exosphere and magnetosphere and provide insights into its surface composition^15–17^.

Mercury’s vicinity has previously been observed in situ by three Mariner flybys in the 1970s^18^ and by NASA’s MESSENGER^19^ mission, which orbited the planet from 2011 to 2015 on an elongated elliptical orbit with a periapsis of 120 km and an apoapsis of 15,000 km from the surface. The Mariner spacecraft achieved a closest approach (CA) distance of 327 km to Mercury, whereas MESSENGER’s CA during a flyby occurred on 29 September 2009, at an altitude of 228 km. Towards the end of its mission, the spacecraft’s orbit was gradually decayed, bringing it progressively closer to Mercury’s surface before it finally impacted the planet. The BepiColombo mission^20^, consisting of two spacecraft, the ESA Mercury Planetary Orbiter (MPO) and JAXA Mercury Magnetosphere Orbiter (MMO), is designed to comprehensively study Mercury, including its geology, interior, magnetic field, surface, exosphere and magnetosphere. Launched on 20 October 2018, BepiColombo is currently in its cruise phase and will be inserted into orbit around Mercury in November 2026. During its journey through the inner heliosphere, BepiColombo has performed multiple flybys of the Earth, Venus and Mercury.

On 4 September 2024, BepiColombo performed its fourth flyby of Mercury, reaching an altitude of only 165 km above the planet’s surface, the closest gravity-assisted manoeuver to a planet. This approach was also clearly closer than the MPO’s planned initial closest point of approximately 480 km once in orbit. During the flyby, Mercury was immersed in enhanced fluxes of energetic particles from the Sun. These particles were detected by the Solar Intensity X-Ray and Particle Spectrometer (SIXS)^21^ instrument onboard MPO, which provides greater spectral sensitivity, better directionality and broader coverage of SEP-relevant electron and proton energies compared with the previous Mercury missions. The combination of this exceptionally close flyby during a period of high solar activity and BepiColombo’s substantially improved SEP detection capabilities, presents a unique opportunity to investigate penetration of energetic particles into the planetary environment.

## Results

### Event overview

**Fig. 1:**
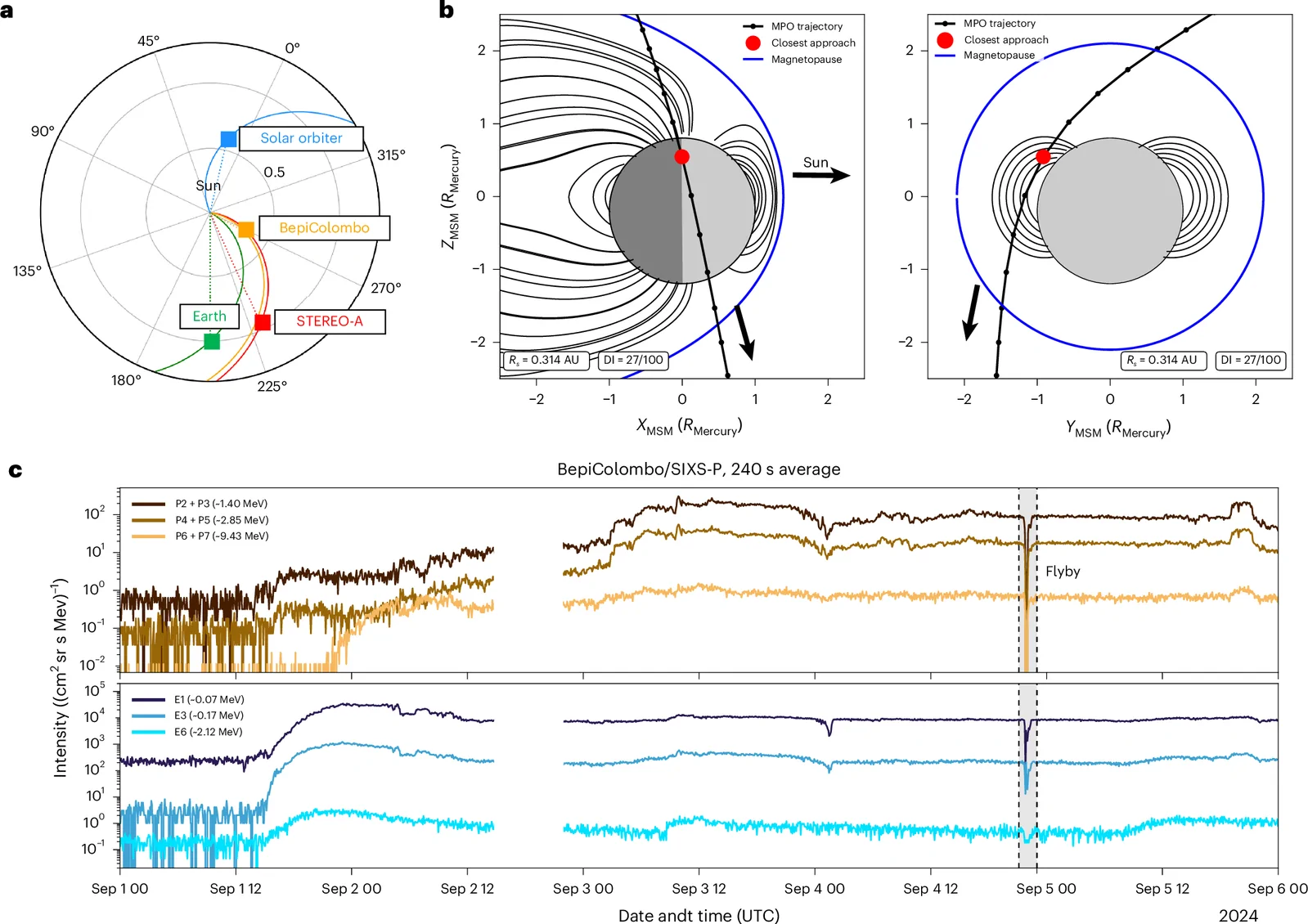
Overview of the BepiColombo 4th Mercury flyby. **a**, Location of BepiColombo, STEREO-A, Solar Orbiter and Earth on 4 September 2024 compiled using Solar-MACH^67^. **b**, Trajectories of BepiColombo’s fourth Mercury flyby on 4 September 2024 in the *X*_MSM_–*Z*_MSM_ (left) and *Y*_MSM_–*Z*_MSM_ (right) planes in units of Mercury radii (2,439.7 km). The thicker black line corresponds to BepiColombo’s trajectory with the black data points corresponding to the spacecraft’s location at intervals of 6 min, and the black arrow shows the direction of travel. The blue line in each panel represents a model magnetopause and the black lines correspond to the modelled average magnetic field calculated using the KTH22 model. **c**, BepiColombo SIXS-P proton (top) and electron (bottom) flux measurements for the selected energy channels. The proton channels combined in pairs of two consecutive channels to increase statistics. The flyby interval is bordered by a pair of dashed lines and shaded grey, to the right of the plot.

**Extended Data Fig. 1:**
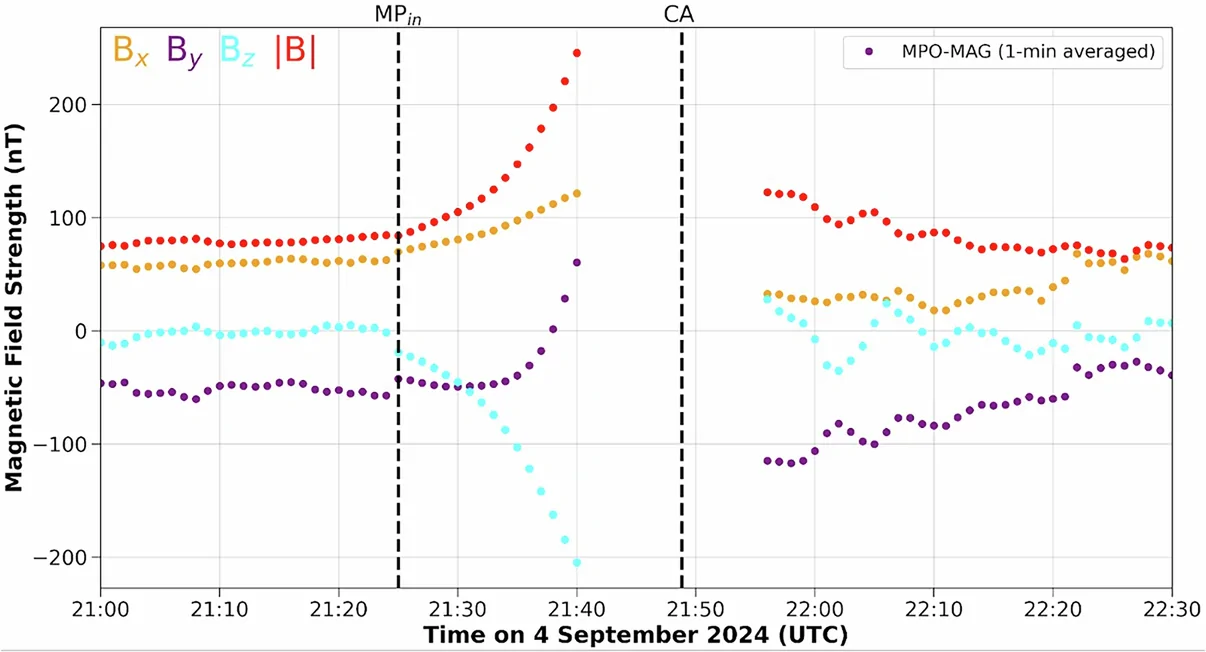
MPO / MAG magnetic field observations. BepiColombo MPO/MAG observations during the planetary flyby were analyzed at a 1-minute cadence using data that are still undergoing calibration. Due to constraints of the cruise phase, the measurements may be subject to offsets. The first dashed line shows the inbound magnetopause crossing.

During this flyby, Mercury’s distance from the Sun was 0.315 astronomical units (AU). Figure 1a shows the location of the Earth, BepiColombo, Solar Orbiter and STEREO-A spacecraft at the time of the flyby. BepiColombo entered the Hermean environment through its northern magnetosphere, tailwards of the cusp, and exited through the dayside southern magnetosphere (Fig. 1b). The magnetic field is taken from the Korth–Tsyganenko–Heyner (KTH22) modular Mercury magnetic field model^22–24^, using a disturbance index (DI) value of 27. This value indicates moderately disturbed magnetospheric conditions and was obtained by optimizing the model to match the inbound magnetopause crossing observed by the MPO magnetometer (Methods; Extended Data Fig. 1).

Figure 1c shows the selected channels representing energetic proton (~1–9 MeV) and electron (~70 keV to 2 MeV) fluxes, measured by the SIXS particle detector (SIXS-P)^21^ instrument onboard BepiColombo/MPO (Methods). These fluxes began to enhance on 1 September 2024, at approximately 15:00 UT, with further enhancements occurring on 3 September 2024. During this period, the Sun was close to its activity maximum, and multiple prominent fast CMEs were detected by the coronagraphs onboard the SOHO (located at the Lagrangian Point L1, about 1.5 million kilometres from the Earth towards the Sun) and STEREO-A spacecraft. In addition, several intense X-ray flares were observed by both the Geostationary Operational Environmental Satellites (GOES) and the Solar Orbiter/STIX instrument, which at the time was positioned almost on the opposite side of the Sun to Earth.

The energetic particle fluxes at BepiColombo remained elevated and steady as the spacecraft approached Mercury. A clear and abrupt decrease in both electron and proton fluxes was observed late on 4 September, coinciding with BepiColombo’s CA to the planet. After this flyby, fluxes recovered to their previous elevated levels, indicating that the decrease was caused by the flyby itself, rather than by subsequent solar activity variations.

The SEPs follow approximately interplanetary magnetic field lines, which generally have relatively low curvature. However, scattering from irregularities in the solar wind and magnetic field leads to cross-field transport, causing their directions to spread over a wide range of angles. As a result, Mercury is exposed to energetic particles arriving from multiple directions rather than from a single field-aligned beam.

### Overview of energetic electron and proton variations

**Fig. 2:**
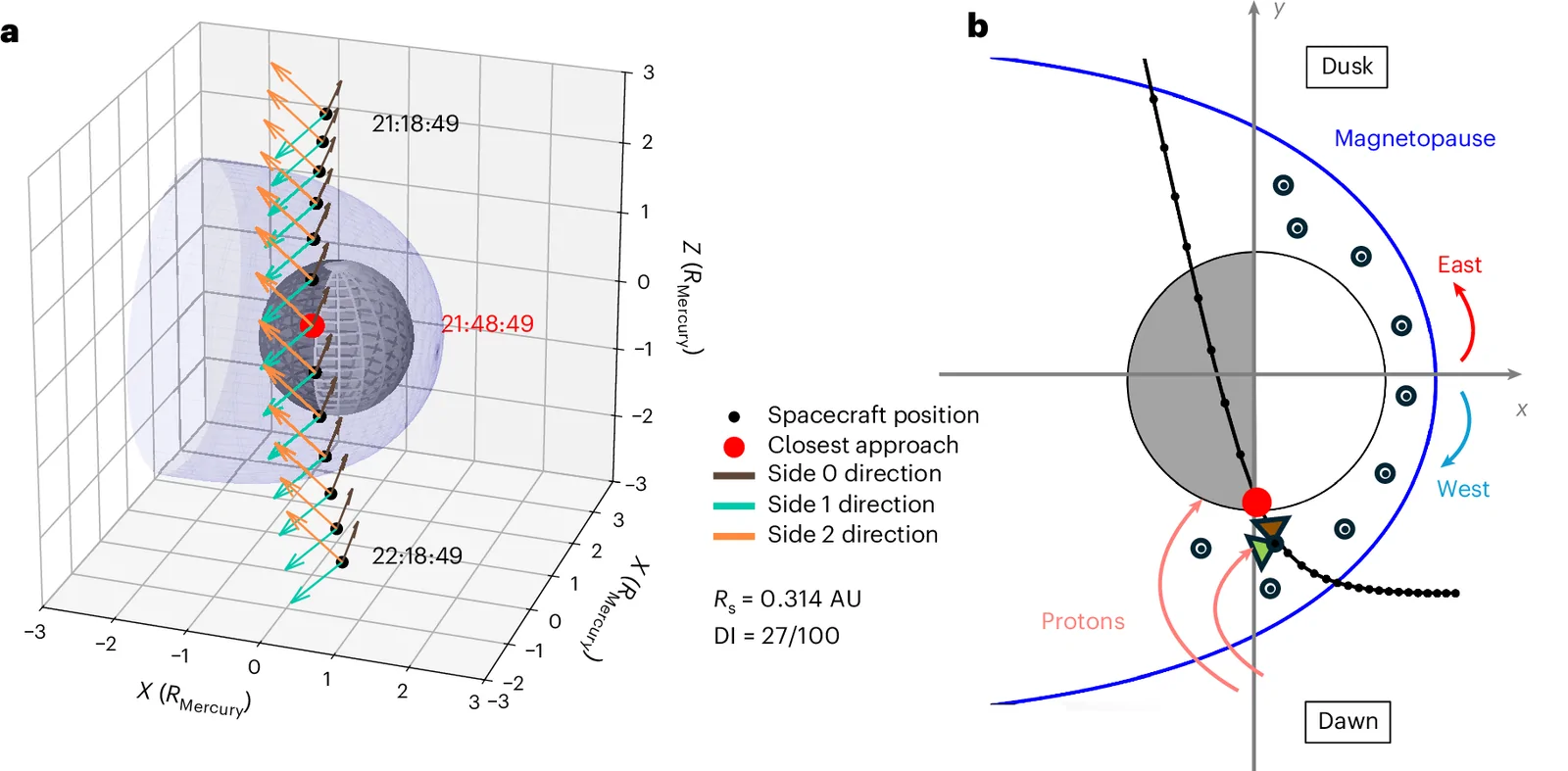
SIXS-P detector side orientations. **a**, Three-dimensional visualization of the orientation of the boresight vectors for SIXS-P detector sides 0, 1 and 2. The light-blue shaded region represents a magnetopause boundary modelled with the KTH22 model for a distance from the Sun of 0.315 AU and a DI of 27/100. The day and night sides of the planet are shown as well as the spacecraft’s position (black dots) at intervals of 6 min. The direction of the boresight vectors for sides 0, 1 and 2 detectors are shown by the brown, green and orange arrows, respectively. **b**, Plot of the BepiColombo trajectory in the *X*–*Y* plane showcasing the westward (clockwise) and eastward (anti-clockwise) directions, as well as the proton gyration direction in the Mercury’s magnetic field. Side 0 points towards the planet, and sides 1 and 2 point approximately tailwards.

During the flyby, the SIXS-P instrument measured energetic particle fluxes using three of its five detectors, each having a viewing cone of approximately 25° wide (Methods). Side 0 was directed towards Mercury, while side 1 and side 2 were oriented upwards and downwards, respectively, and both viewing westwards (Fig. 2).

**Fig. 3:**
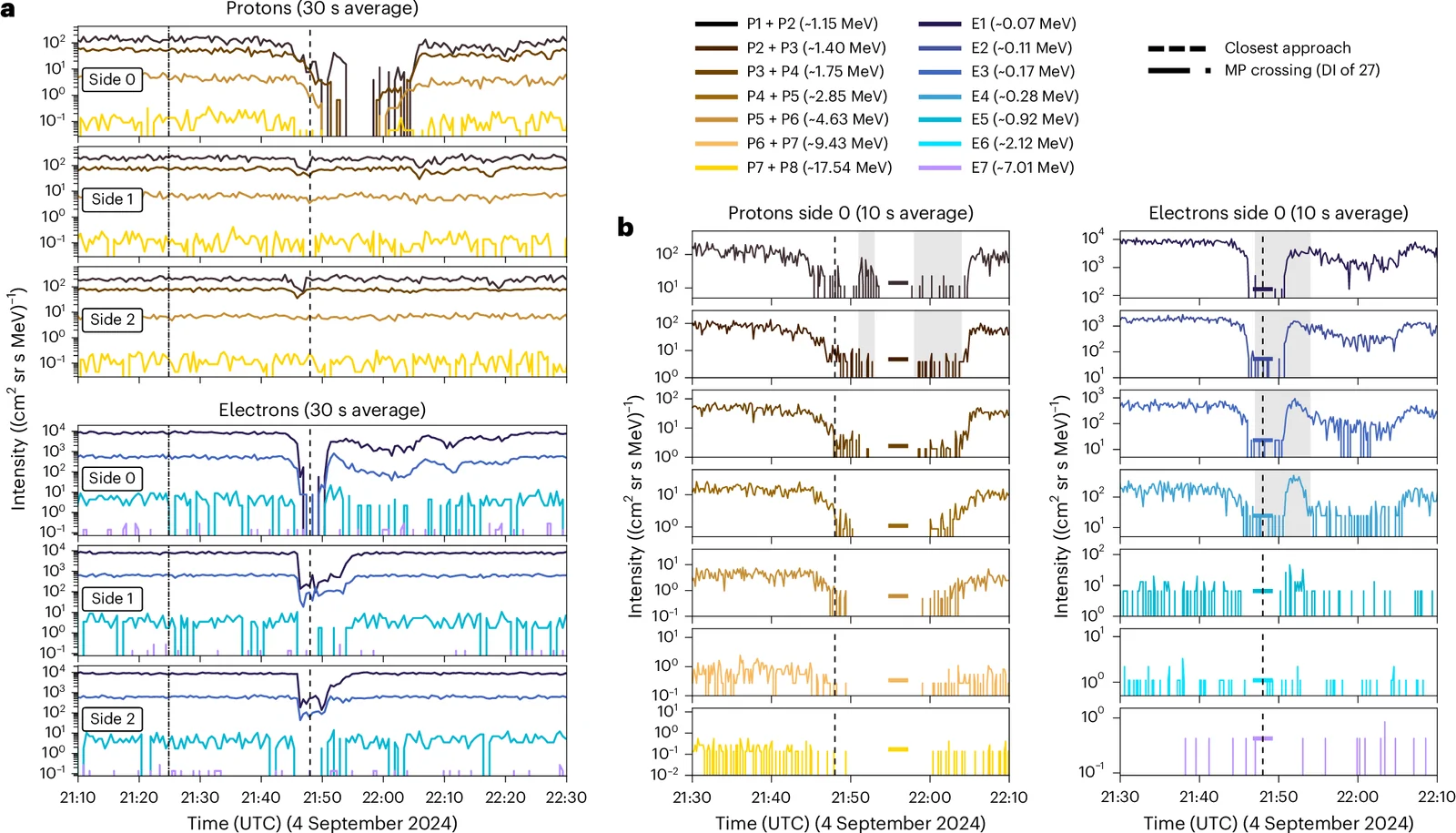
SIXS-P Proton and electron flux measurements. **a**, Proton (combined channels P1 + P2, P3 + P4, P5 + P6 and P7 + P8) and electron fluxes (channels E1, E3, E5 and E7) during BepiColombo’s fourth Mercury flyby on 4 September 2024 with 30-s averaging of the data. **b**, Combined proton channels from P1 + P2 to P7 + P8 and electron channels from E1 to E7 as 10-s averages shown separately for side 0 (corresponding plots for sides 1 and 2 are given in Extended Data Figs. 2 and 3). The horizontal bars the one-count levels, representing the instrumental detection threshold. The grey shaded regions show the intervals when the lowest side 0 proton channels are contaminated by electrons (Methods). The dashed line shows the time of CA at 21:48 UT, and the dashed-dotted lines the inbound and outbound magnetopause (MP) crossing times from the KTH22 model with DI of 27.

**Extended Data Fig. 2:**
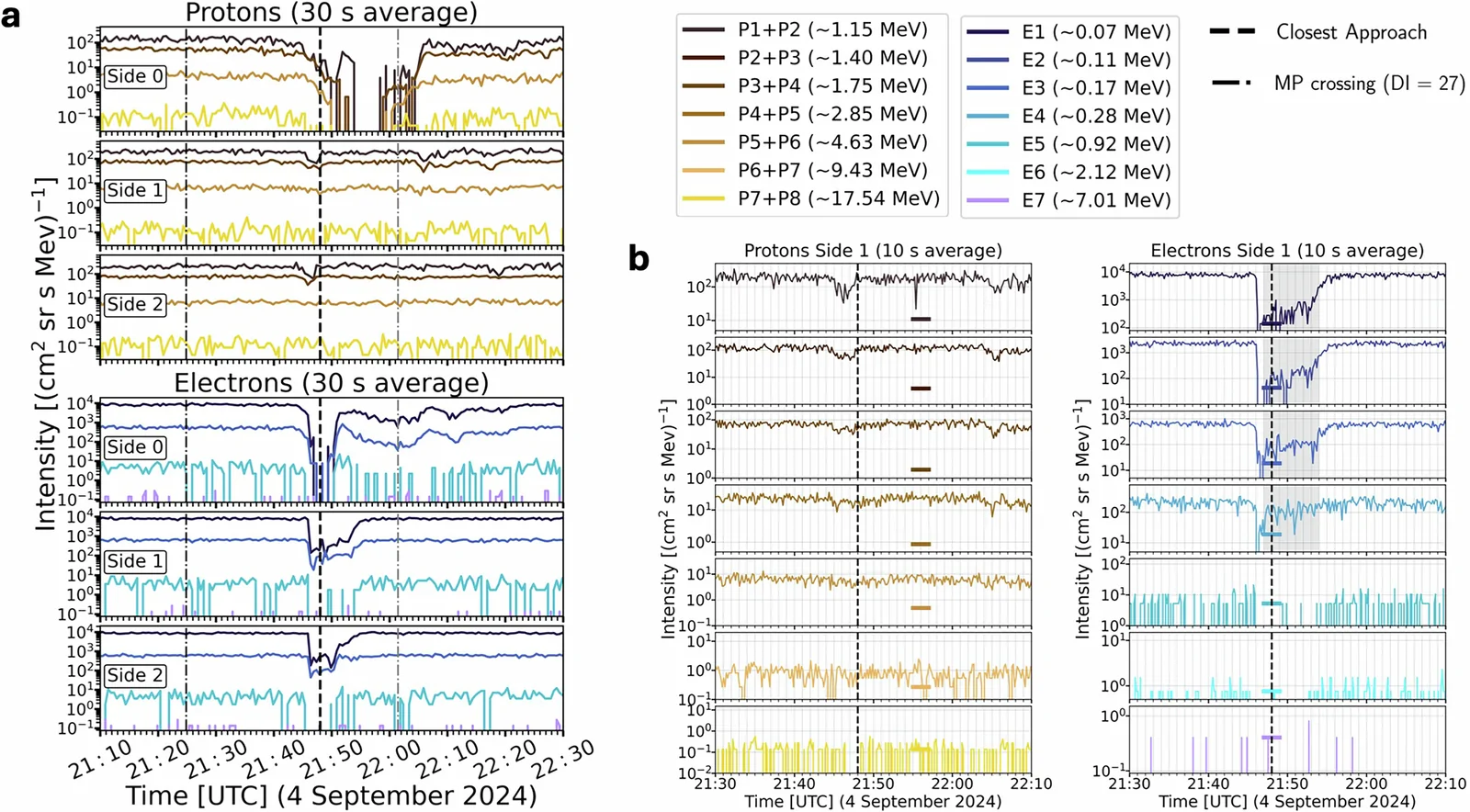
SIXS-P proton and electron flux measurements for Side 1. **a**, Proton (combined channels P1+P2, P3+P4, P5+P6, and P7+P8) and electron fluxes (channels E1, E3, E5 and E7) during BepiColombo’s fourth Mercury flyby on 4 September 2024 with 30-second averaging of the data. **b**, Combined proton channels from P1+P2 to P7+P8 and electron channels from E1 to E7 as 10-second averages shown separately for Side 1. The horizontal bars in each panel show the one-count levels, representing the instrumental detection threshold. The dashed line shows the time of closest approach at 21:48 UT, and the dashed-dotted lines the inbound and outbound magnetopause (MP) crossing times from the KTH22 model with DI = 27.

**Extended Data Fig. 3:**
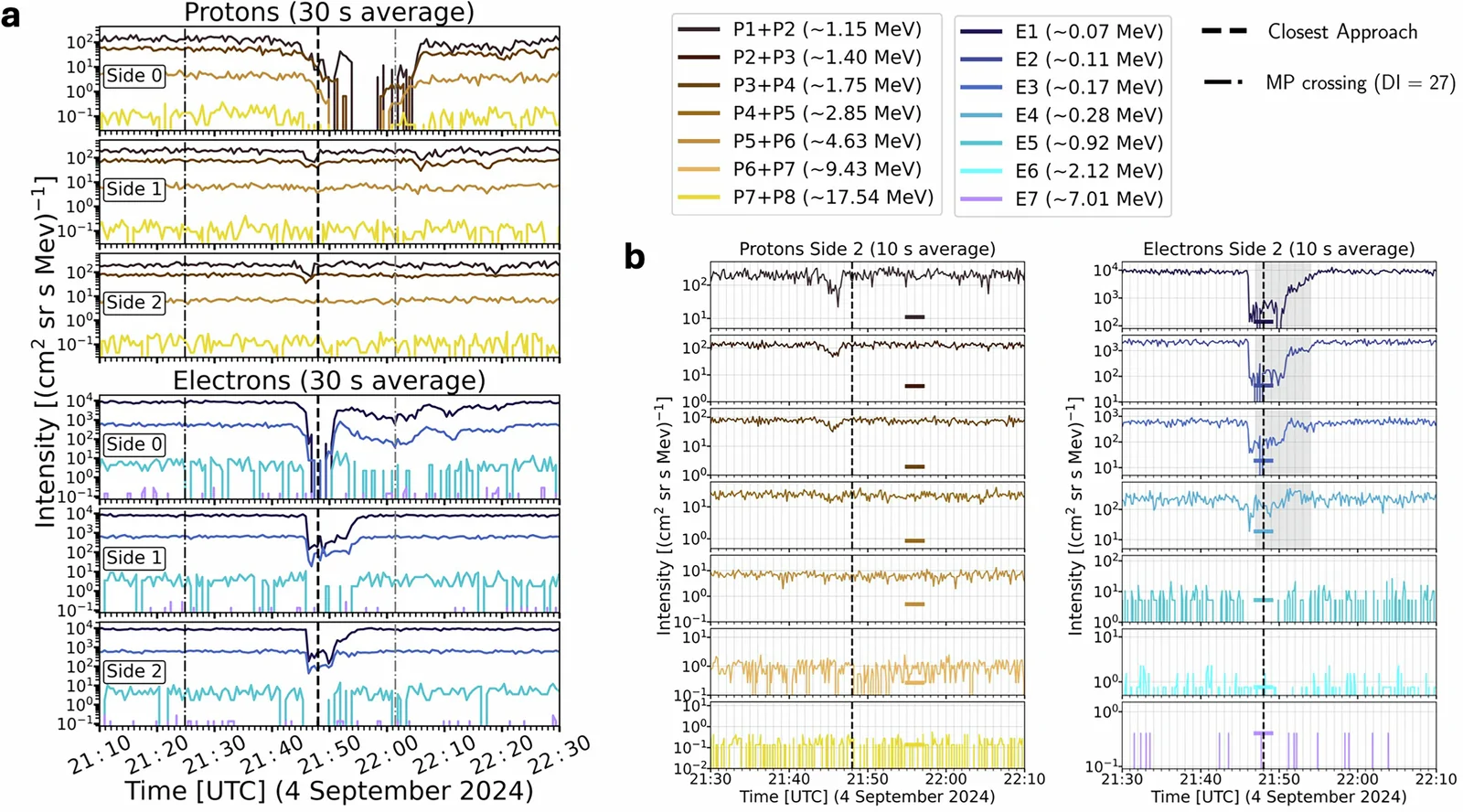
SIXS-P proton and electron flux measurements for Side 2. **a**, Proton (combined channels P1+P2, P3+P4, P5+P6, and P7+P8) and electron fluxes (channels E1, E3, E5 and E7) during BepiColombo’s fourth Mercury flyby on 4 September 2024 with 30-second averaging of the data. **b**, Combined proton channels from P1+P2 to P7+P8 and electron channels from E1 to E7 as 10-second averages shown separately for Side 2. The horizontal bars in each panel show the one-count levels, representing the instrumental detection threshold. The dashed line shows the time of closest approach at 21:48 UT, and the dashed-dotted lines the inbound and outbound magnetopause (MP) crossing times from the KTH22 model with DI = 27.

A closer examination of the SIXS-P measurements around the time of the CA at 21:48 UT reveals a clear directionality in the particle flux response and notable differences in the electron and proton trends (Fig. 3; see Extended Data Figs. 2 and 3 for separate side 1 and side 2 electron and proton fluxes). The most dramatic decreases are observed by side 0.

The side 0 proton fluxes begin to decrease gradually around 21:43 UT. Near the CA, they drop sharply and finally disappear across all energy channels. The grey shaded enhancements at the lowest proton energies observed during the dropout is discussed in detail later. By contrast, on sides 1 and 2 the proton fluxes remain relatively stable throughout the flyby, showing only minor reductions before the CA.

The electron fluxes, on the other hand, exhibit an abrupt decrease on all investigated sides and energies starting around 21:46 UT, a few minutes before the CA. The fluxes recover sharply on side 0 approximately 2 min after the CA (~21:50:30 UT), although relatively large variations continue beyond the marked magnetopause crossing time. By contrast, the fluxes observed by sides 1 and 2 recover more gradually but stabilize relatively early to resemble profiles preceding the dropout.

### Proton flux variations and planetary shadowing

**Fig. 4:**
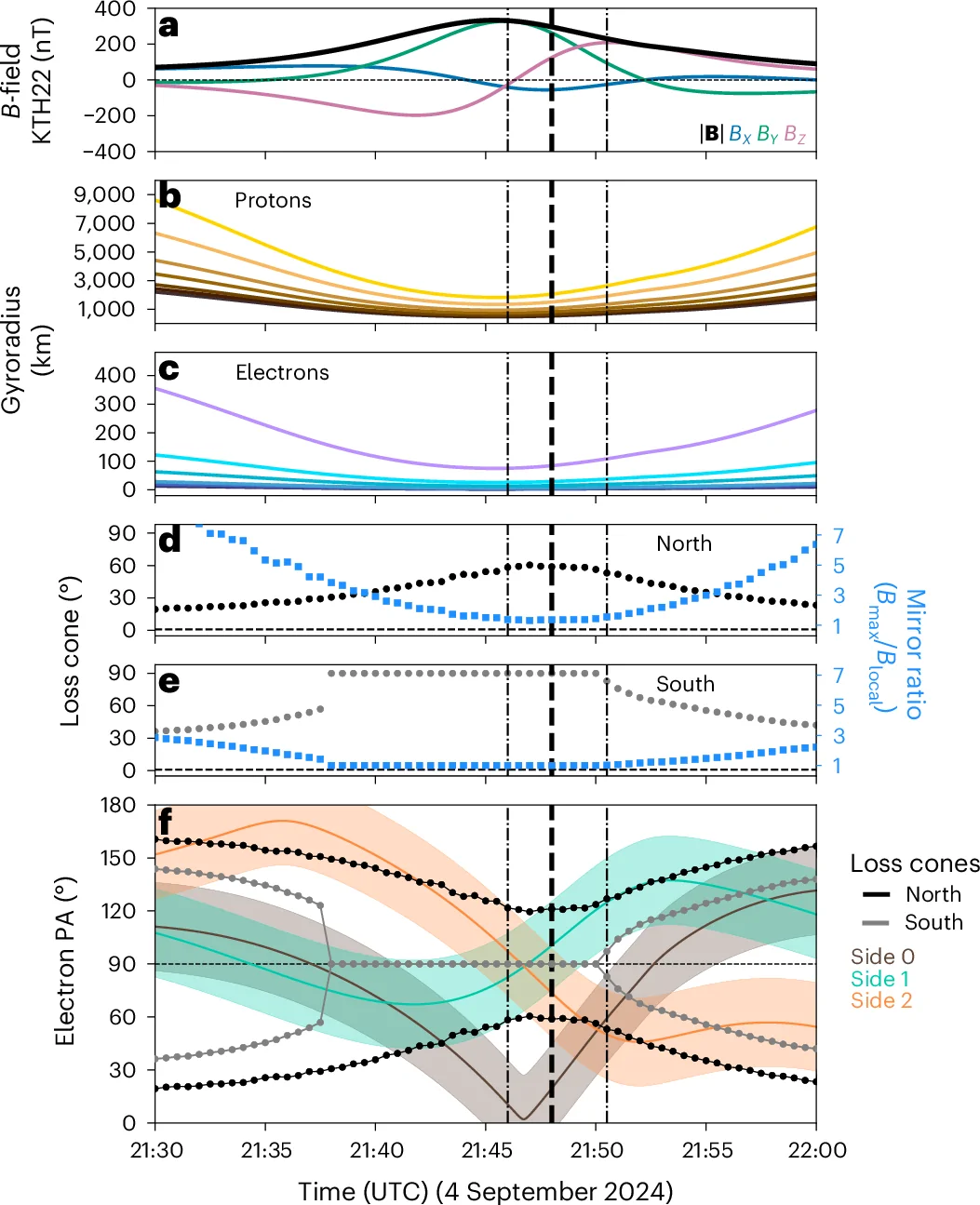
Particle motion in Mercury’s magnetic field. **a**, Magnetic field magnitude and components estimated from the KTH22 model. *B* field, the magnetic field in general; |**B**|, the magnitude of the magnetic field, taken from the vector field. **b**,**c**, Proton and electron gyro radii calculated for SIXS-P energies (Fig. 3). **d**,**e**, Loss cone critical angle (black and grey circles) and the mirror ratio (blue squares) for the northern and southern hemispheres, calculated using the KTH22 model. The mirror ratios are defined as the ratio of the magnetic field strength along the local field line (towards north and south from the spacecraft location, respectively) and the field magnitude at the spacecraft’s position. **f**, Electron PAs with the shaded areas showing the 25° viewing cones and with the northern and southern loss cone widths shown by black circles and grey squares, respectively. The PAs are defined as the angle between the boresight unit vector for each detector and the magnetic field unit vector. They are obtained from the KTH22 model instead of direct measurements due to calibration issues during the cruise phase, when MPO and MMO are stacked with the Mercury Transfer Module (MTM) in a magnetically unclean configuration. The dashed thick vertical line indicates the CA and thinner vertical lines the strongest electron dropout.

The well-known east–west effect, in which charged particles are bent by the planetary magnetic field, can lead to directional differences in the detected fluxes, for example, refs. ^25,26^. In Mercury’s magnetic field, energetic protons have relatively large gyroradii, comparable to the gradient/curvature scales of its magnetosphere (Fig. 4b). For example, a 2-MeV proton in a 200-nT magnetic field would have a gyroradius of approximately 1,000 km. Therefore, these particles cannot be trapped within Mercury’s magnetosphere but instead impact to the surface in a process referred to as planetary shadowing. Shadowing may also arise from purely magnetic deflection, in which high-energy particle trajectories are bent away from the planet above the detector, for example, ref. ^27^. However, for ≳1-MeV protons, planetary shadowing is expected to dominate, because their paths are only weakly bent by the Mercury’s magnetic field.

**Fig. 5:**
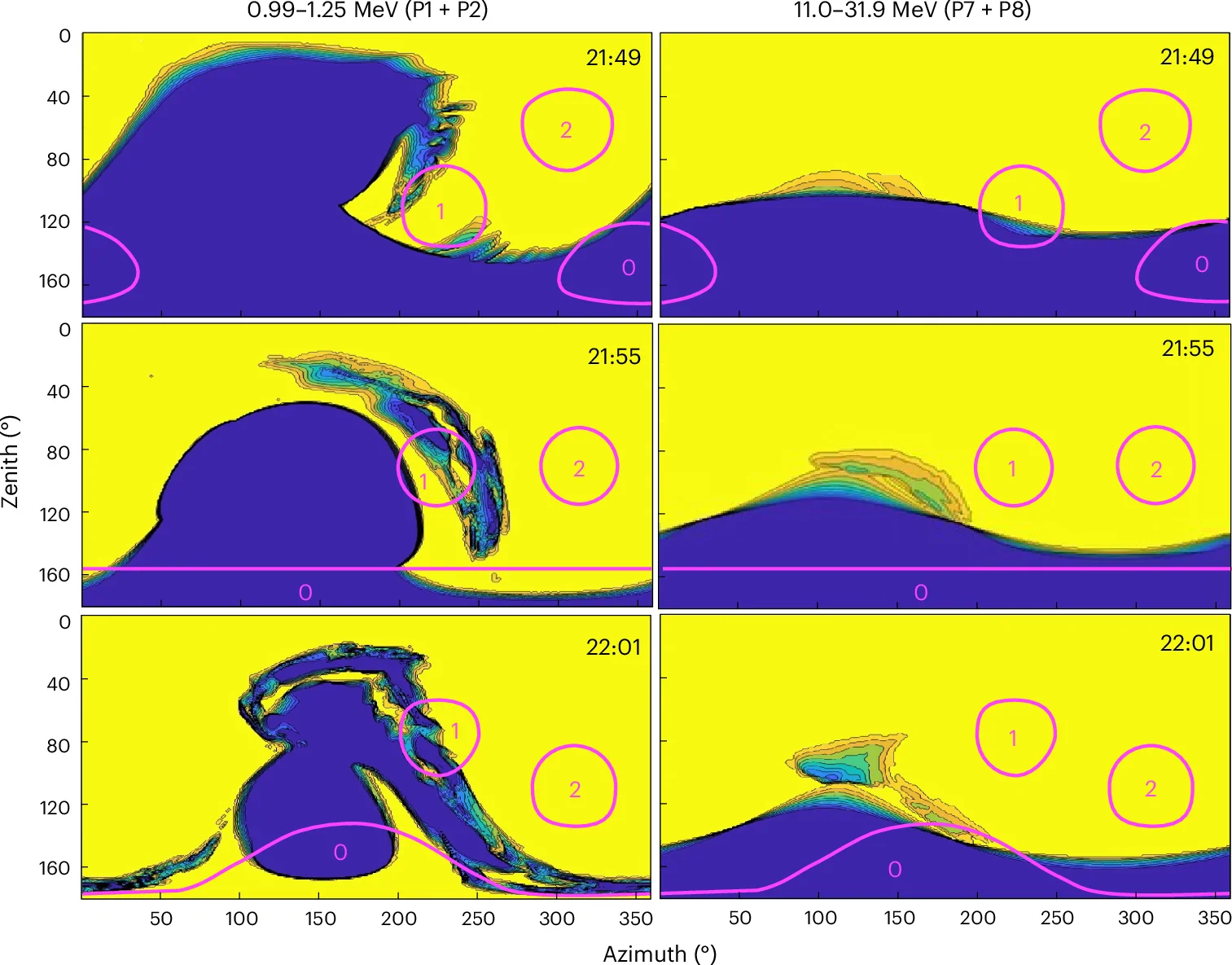
Modelled allowed and forbidden proton trajectories. Contour plots of 0.99–1.25-MeV (SIXS-P P1 + P2) and 11.0–31.9-MeV (SIXS-P P7 + P8) protons that have been simulated using the Toffoletto–Hill field model with backwards tracing from the spacecraft’s position at 21:49, 21:55 and 22:01 UT. The coordinates are the local zenith/azimuth at the spacecraft position and the azimuth is measured from the local horizontal north towards the east. The yellow colour indicates allowed trajectories, that is, particles that could have accessed the detectors from interplanetary space, whereas the blue colour indicates forbidden trajectories, that is, particle trajectories that would intersect the planet. The planetary shadow and a penumbra (a chaotic region) between the allowed and the forbidden trajectories (greenish colour) is also seen. Three view cones (sides 0, 1 and 2) of SIXS-P are also indicated.

On the dayside near the equatorial region, Mercury’s magnetic field points northwards (upwards), causing positively charged protons to gyrate clockwise when viewed above, as depicted in Fig. 2. Consequently, more protons are expected arriving from the west than from the east. The tailward-facing SIXS-P detectors (side 1 and side 2) should therefore capture protons with minimal obstruction. By contrast, Mercury-facing side 0 is expected to exhibit large dropout as protons may intercept the planet’s surface before their path curves enough to reach the detector. These expectations are in agreement with the observations presented in Fig. 3. The small flux reductions on sides 1 and 2 before the CA are probably due to partial obscuration of the detector by the planet combined with the wide angular width of their viewing cones (half-width of approximately 25°). The above described expectations and observations are also in agreement with the Toffoletto–Hill field modelling^28^ of allowed (yellow regions) and forbidden (blue regions) proton trajectories, shown in Fig. 5; side 0 corresponds largely forbidden trajectories that intersect the planet, whereas sides 1 and 2 are on allowed orbits meaning the protons can reach the detector sides without intersecting the planet.

**Extended Data Fig. 4:**
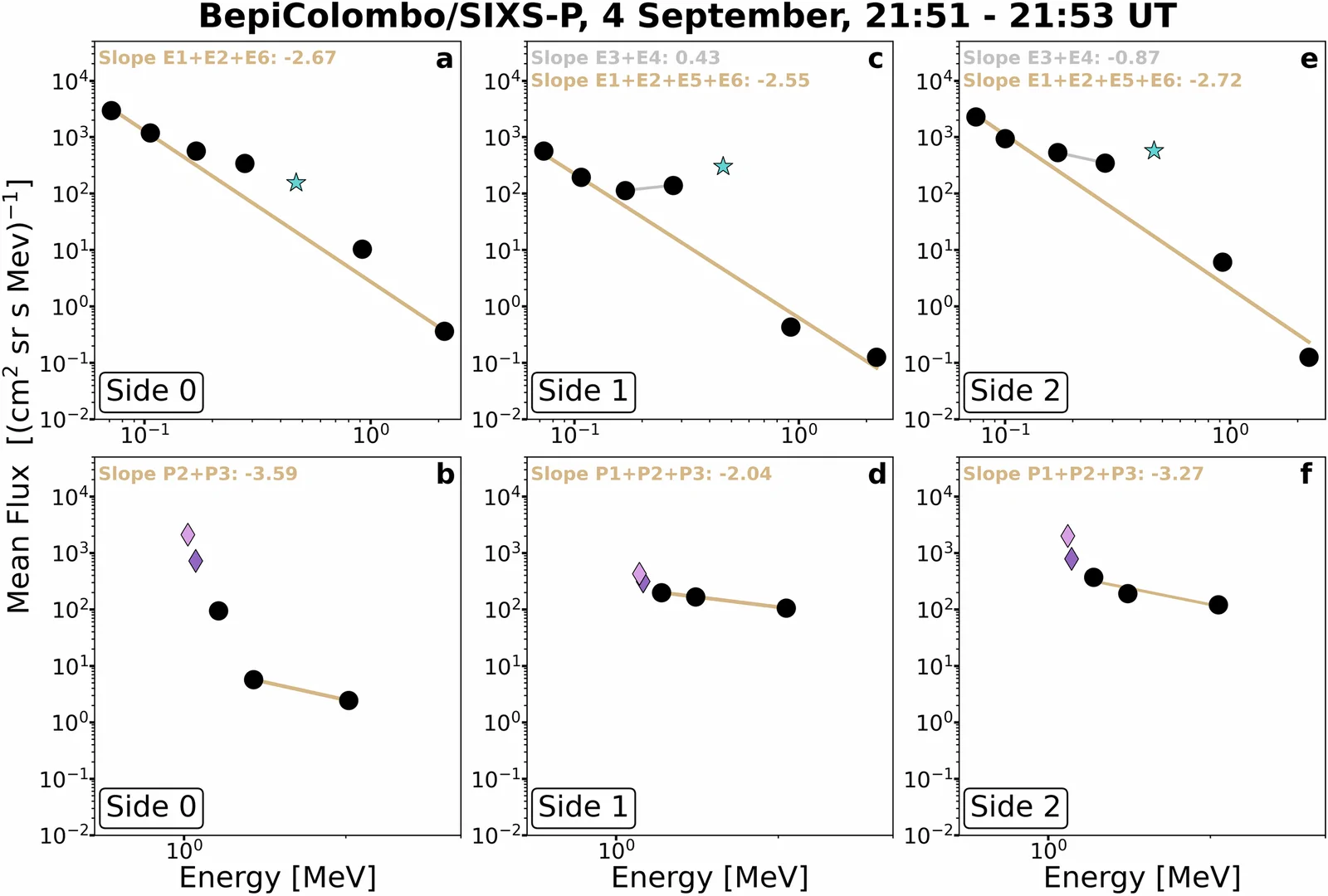
Energy spectra during Side 0 proton dropout. The top row shows the electron spectra calculated over the period from 21:51 – 21:53 UT for channels from E1 to E6 (black circles) and Sides 0, 1 and 2. Blue stars represent the proton P1 channel that has been converted to an electron channel. The fits are calculated for the selected electron channels. The bottom row shows the proton spectra calculated for the same period from P1 to P3 (black circles) and Sides 0, 1 and 2. Purple diamonds represent electron channels E3 and E4 that have been converted to proton channels. The fits are calculated for the selected protons channels.

On side 0, higher-energy protons reach the one-count levels earlier, as their larger gyroradii allow them to intersect the planet sooner. The enhancement observed in the lowest energy proton channels between 21:51 and 21:53 UT (Fig. 3, grey shaded area) is due to electron contamination; during this interval, the P1 proton channel, when converted to an equivalent electron flux, closely matches the measured electron energy spectra (Methods; Extended Data Fig. 4). Detections before this interval are, however, real protons counts as they occur during a complete electron dropout.

The fluxes recover earlier for higher-energy than for lower-energy protons, whereas the Toffoletto–Hill simulation shows higher-energies to have a larger fraction of forbidden trajectories at this time. Although higher-energy protons are generally expected to impact the planet for longer, the delayed recovery observed for lower-energy protons reflects their greater sensitivity to the magnetic field; due to their smaller gyroradii, they are more easily bent by the field and prone to effects related to field curvature and small-scale magnetic variations, leading to enhanced drifts and pitch-angle scattering, for example, ref. ^29^. The discrepancy between the model and observations therefore probably arises from the limitations of the Toffoletto–Hill magnetic field formulation in accurately producing the magnetopause and field geometry near the dawn-flank.

### Origin and variations of high-energy electrons

During the investigated period, the electrons observed by SIXS-P are expected to be primarily of solar origin that have become quasi-trapped within Mercury’s magnetic field, rather than being produced by internal magnetospheric processes such as substorms^30–34^. Due to their smaller gyroradii (Fig. 4c), electrons originating from the Sun/solar wind can only access Mercury’s magnetosphere either through the polar cusps or field lines opened by magnetic reconnection between the interplanetary magnetic field and Mercury’s magnetic field. Once electrons gain access to the magnetosphere, they start curvature drifting eastwards. Relatively constant electrons fluxes observed during most of the flyby are consistent with Mercury having been exposed to high fluxes of SEPs for several days (Fig. 1), suggesting sufficient time to diffuse, resulting in a stable flux signature.

**Fig. 6:**
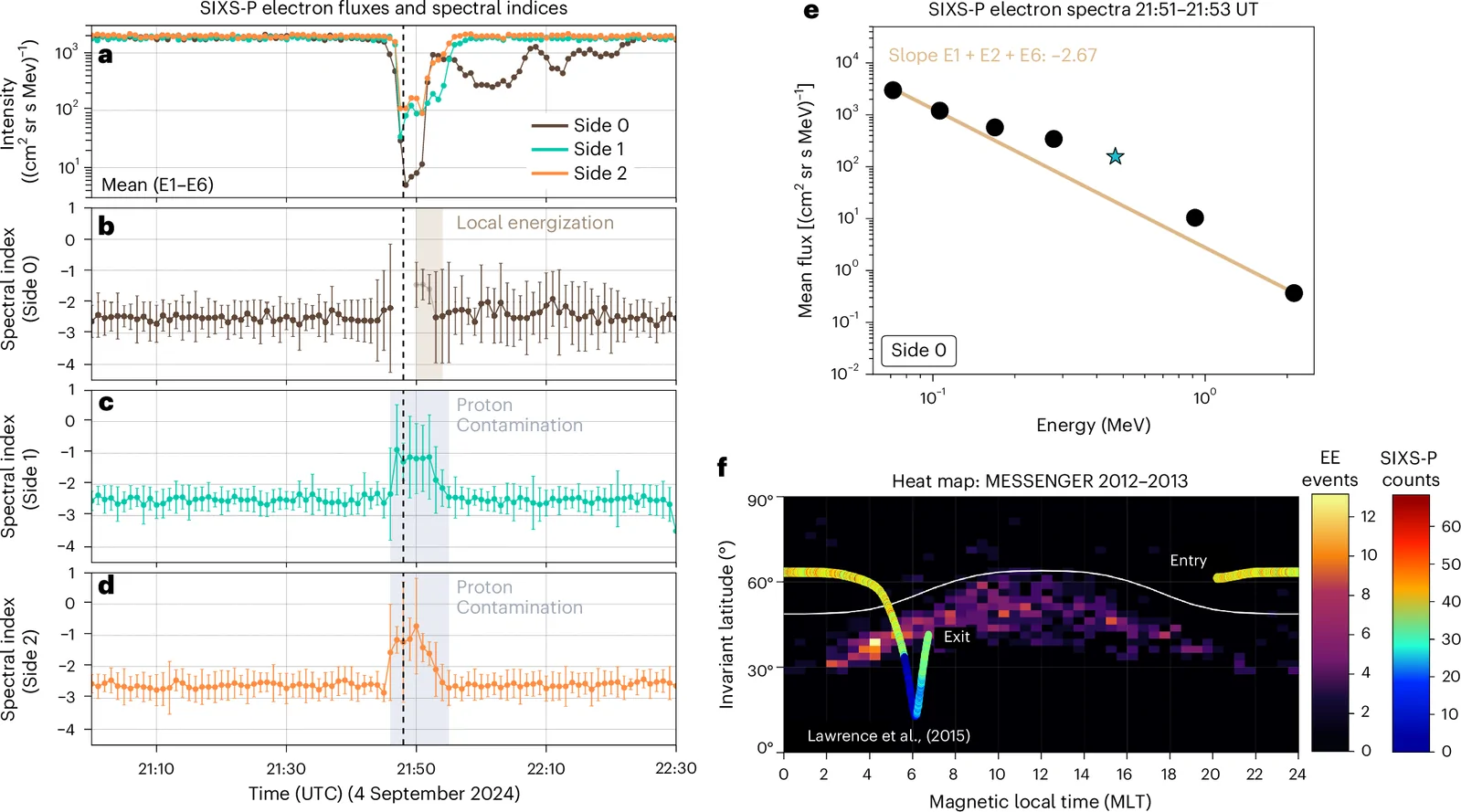
Evidence for solar origin of energetic electrons. **a**–**d**, SIXS-P (**a**) 60-s electron fluxes averaged over channels E1–E6 and spectral indices for sides 0 (**b**), 1 (**c**) and 2 (**d**), averaged from 1-s spectral indices. Error bars indicate the standard deviation of each 60-point sample. The dashed black line represents the time at CA. Side 0 spectral indices between 21:47 and 21:50 UT are excluded due to low counts. The shaded regions indicate intervals where spectral indices are probably affected by local energization (side 0; brown shaded area) and proton contamination (side 1 and 2; blue shaded area). **e**, SIXS-P side 0 electron spectra showing the local energization where the blue star is the P1 channel converted to equivalent electron channel (Methods). **f**, Total SIXS-P electron counts during the fourth Mercury flyby overlaid with a heat map of bursty energetic electron event observed during MESSENGER’s 8-h orbits from April 2012 to December 2013 plotted as a function of invariant latitude and magnetic local time (MLT), following ref. ^39^. The map includes electron events up to 200 keV, corresponding to SIXS-P channels E1–E4. The solid white line denotes the average open-closed field boundary.

Support for the solar origin of these electrons is given by the relatively hard energy spectra with spectral indices typical of SEP events, for example, refs. ^35,36^, as shown in Fig. 6b–d. The indices also remain at similar values for most of the flyby, except around the CA, where they become considerably harder, exhibiting a shallower slope. During this interval, the side 0 energy spectra (Fig. 6e; Methods) reveals an enhancement at energies peaking around ~200−400 keV. When the affected channels from E3 to E5 are excluded, the derived spectral index becomes consistent with the SEP spectra. The pitch angle (PA) range sampled by the side 0 detector coincidentally approaching 90° (Fig. 4f), suggests that the observed electron spectra represent a locally further energized SEP population. At this time, whistler waves were reported in Mercury’s magnetosphere^37^, which, in the Earth space environment, are known to efficiently accelerate electrons to relativistic energies, for example, ref. ^38^. Electrons with PAs close to 90° are expected to be preferentially enhanced by whistler waves, as they remain confined near the magnetic equator where these waves are most intense and frequently observed. Furthermore, the side 0 viewcone lies outside the calculated loss cone at this time (Fig. 4f). By contrast, electrons observed by sides 1 and 2 correspond to more field-aligned PAs, causing them to spend less time in the equatorial region and thus be less affected by whistler waves. Their viewcones also partially intersect the loss cone, allowing a fraction of these electrons to precipitate.

**Extended Data Fig. 5:**
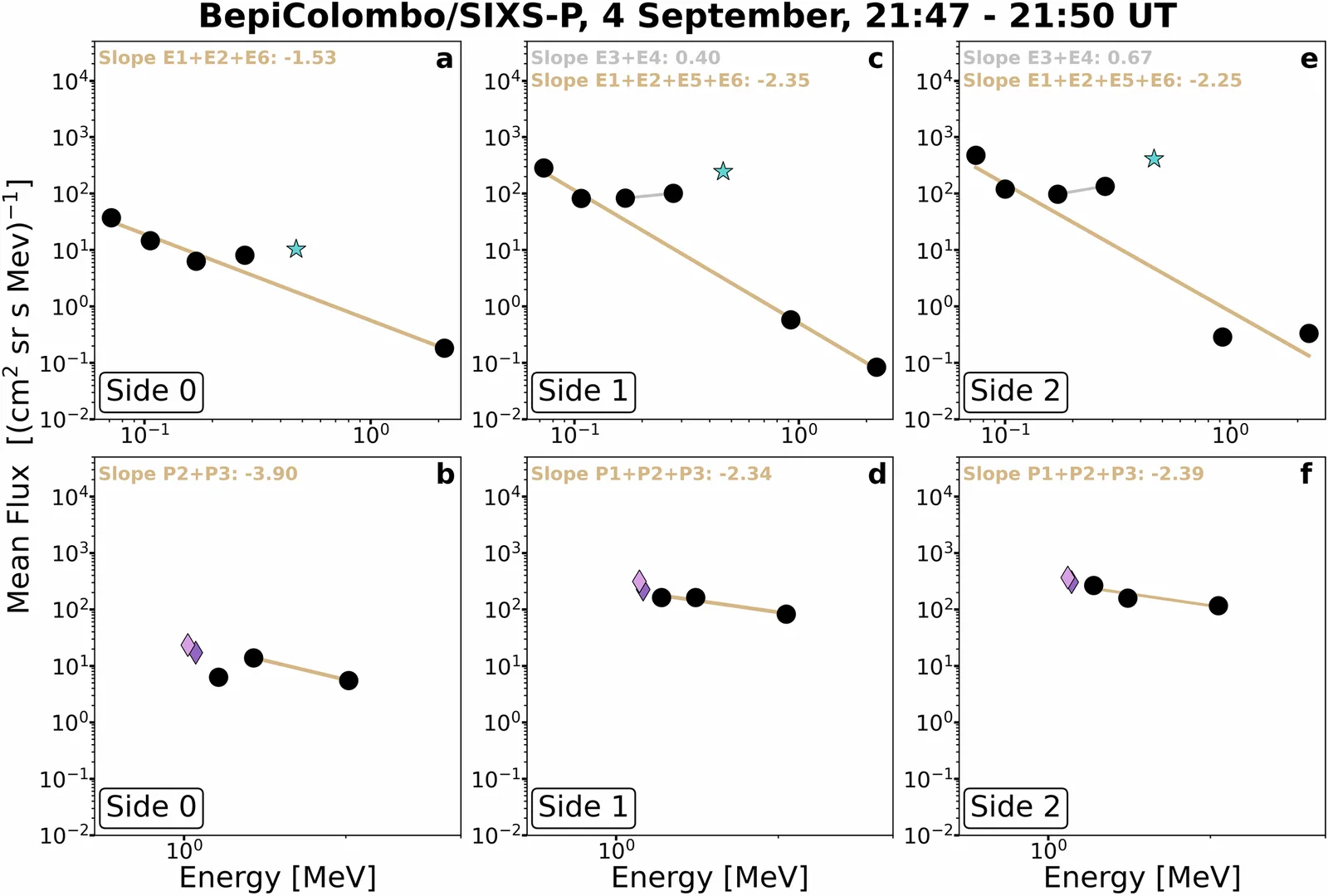
Energy spectra during electron drop-out. The top row shows the electron spectra calculated over the period from 21:47 - 21:50 UT for channels from E1 to E6 (black circles) and Sides 0, 1 and 2. Blue stars represent the proton P1 channel that has been converted to an electron channel. The fits are calculated for the selected electron channels. The bottom row shows the proton spectra calculated for the same period from P1 to P3 (black circles) and Sides 0, 1 and 2. Purple diamonds represent electron channels E3 and E4 that have been converted to proton channels. The fits are calculated for the selected protons channels.

However, the harder spectral indices on side 1 and 2 are the result of proton contamination (note that unlike on side 0, the proton fluxes on these sides did not drop). These detectors observed high proton fluxes throughout the event and converting the affected electron channels to equivalent proton fluxes shows that they align well with the proton energy spectra around the CA (Extended Data Fig. 5). The converted electron channels lying slightly above the proton energy spectra suggest the presence of a minor electron population, probably accelerated by whistler-mode waves, also detected on sides 1 and 2. The proton contamination also explains why the electron fluxes on sides 1 and 2 do not decrease as low as on side 0.

Further evidence of solar origin comes from a comparison of SIXS-P observations with a statistical 8-h orbit map of MESSENGER high-energy electron bursts, in which SEP events were excluded and only electrons accelerated during magnetospheric substorms were selected (Fig. 6f). During its fourth flyby, BepiColombo observed elevated electron counts over a considerably broader region than where MESSENGER detected electron bursts (with 12-h orbit map^39^ the overlap is even smaller).

Electrons cannot be shadowed by the planet through the same mechanism as protons, as they circle in the opposite direction (east to west) and have substantially smaller gyroradii. Instead, the dropout may occur when their PAs are within the local loss cone (Methods). In the Hermean magnetosphere, these conditions must be evaluated separately for the two hemispheres, as Mercury’s magnetic field is much weaker in the southern hemisphere than the northern hemisphere^40,41^.

The northern loss cone half-width reached a maximum of about 60° close to CA, whereas the southern loss cone spans 90° between 21:38 and 21:50 UT (Fig. 4d–e), implying that all PAs should precipitate. However, for most of the periods when PAs are predominantly within the loss cone, the electron fluxes remain relatively steady. A possible explanation is that precipitation does occur, but the field lines are opened by magnetic reconnection, allowing electrons to be continuously replenished by the ongoing SEP event. This interpretation is consistent with the observed SEP-like spectral indices and with reconnection at the Hermean magnetopause being a common process for nearly all interplanetary magnetic field orientations, for example, ref. ^42^. The onset of the electron dropout occurs slightly below the invariant latitudes at which MESSENGER ceased observing energetic electron bursts. This suggests that SEP electrons accessing along reconnection-opened field lines are also limited from the innermost regions and, if some do enter, they precipitate rapidly.

## Conclusion

We have analysed observations from the fourth Mercury flyby of the BepiColombo mission on 4 September 2024. During this flyby, BepiColombo approached only within 165 km of Mercury’s surface, whereas the planet was bathed in high fluxes of energetic electrons and protons from the Sun. These unique observations from the SIXS-P particle detector, combined with modelling of allowed and forbidden proton trajectories, provide direct evidence for planetary shadowing of high-energy protons at Mercury. Although the sharp electron dropout is likely associated with changes in magnetic topology, the presence of a wide loss cone and sustained fluxes during ongoing SEP activity suggests continuous precipitation over a large fraction of the flyby. We further present electron spectra indicative of local acceleration of an SEP population within the Hermean magnetosphere by whistler-mode waves. The spectra showed PA and energy dependent features consistent with whistler wave acceleration, that is, enhancement peaking around hundreds of keV close to 90° PAs.

The study of the access of energetic particles to Mercury’s surface is highly important for understanding space weathering, which refers to physical and optical changes on the surfaces of atmosphereless celestial bodies, including planets, moons, asteroids and comets, induced by the harsh space environment^43,44^. Due to their higher energies, possibly further increased by local acceleration, SEPs can penetrate deeper into the regolith, enhancing effects on radiation damage and mineral structure alteration such as formation and modification of nanophase iron and amorphization. Sustained SEP precipitation during periods of high solar activity may therefore produce long-term effects, potentially leading to solar-cycle-dependent variations in surface and exospheric properties. Furthermore, for a planet with an offset dipole magnetic field such as Mercury, these effects are expected to be hemispherically asymmetric^32,45,46^.

The upcoming observations of energetic particles by SIXS, in combination with other BepiColombo instruments, will play an important role in providing new insights into Mercury’s surface processes, and their interplay with the planet’s exosphere and magnetosphere. In particular, the Mercury Imaging X-ray Spectrometer (MIXS)^47^ will measure, once BepiColombo is in orbit around Mercury, X-ray fluorescence^14^ induced by incident energetic particles and X-rays. These joint observations will enable mapping of the elemental composition of Mercury’s regolith and provide key constraints on surface-environment interactions. Furthermore, our careful analysis of cross-talk between particle species is crucial for the future interpretation of SIXS measurements and has broader relevance for energetic particle observations by the current and future planetary and heliospheric missions.

Our results have wide implications in particular to rocky planets close to their parent stars, such as those in the TRAPPIST-1 system^48^. Similarly to Mercury, these planets are expected to be exposed to high fluxes of energetic charged particles. Understanding the shielding mechanisms provided by their potential magnetic fields is crucial for estimating the impact to their environments, including atmospheric chemistry, composition and dynamics^49–51^.

## Methods

### SIXS-P electron and proton measurements

This study uses measurements from the SIXS^21^ onboard the BepiColombo MPO. The primary purpose of the instrument is to investigate X-rays and fluxes of energetic particles incident on Mercury’s surface when BepiColombo is inserted in orbit of the planet (November 2026). BepiColombo was launched in October 2018. During the cruise phase, the SIXS particle detection system (SIXS-P) has been making observations, including during the six flybys of Mercury.

SIXS-P consists of five 150-μm-thick silicon PIN detectors surrounding a cuboid CsI(Tl) scintillator (5 × 5 × 6.3 mm^3^) with a photodiode readout. Three of these five detectors are not obstructed during the cruise phase. Valid particle events in the detector consist of a hit in a single Si detector with all other detectors in anti-coincidence or one Si detector with the scintillator in coincidence and other Si detectors in anti-coincidence. SIXS-P records electrons from 50 keV to ~3 MeV (protons from 1 to ~30 MeV) in six (eight) differential energy channels labelled E1 … E6 (P1 … P8). Electron energy channels E1–E4 (proton energy channels P1–P4) are recorded with a single Si detector and E5–E6 (P6–P8) are recorded as coincidence channels with the scintillator (P5 is a channel with counts from both a single Si detector and coincidence with the scintillator). The Monte Carlo simulated response functions of the channels are used to compute the effective energy of the channel and its corresponding geometric factor that allows one to convert the counting rate in the channel to particle intensity at a given effective energy.

Supplementary Fig. 1 gives the SIXS-P response to electrons and protons for side 0 (with sides 1 and 2 exhibiting nearly identical responses). The upper row presents the response functions to the nominal particle species, whereas the lower row shows the responses to contaminating species. The left column illustrates the responses of the electron (E) and proton (P) channels to electrons, and the right column their responses to protons. Nominal proton channels exhibit near box-car responses, whereas electron channels display more complex response functions, characterized by a nominal energy range and extended high-energy tails. The effective energies of the E, P, PE and EP channels are determined using the bow-tie method, minimizing systematic uncertainties associated with representing each channel by a single effective energy and geometric factor (see ref. ^52^).

### Proton/electron contamination

Because single detector channels do not allow firm particle identification, electron channels E1–E4 can be contaminated by ~1-MeV protons, whereas proton energy channels P1–P2 can be contaminated by >300-keV electrons. Coincidence channels E5–E6 and P6–P8 are clean, because two energy loss measurements allow the determination of the particle species using the d*E*/d*x* versus *E* method.

When an SEP event is ongoing, electrons are often contaminating protons during the event onset, when the >300-keV electrons have already reached the spacecraft but ~1-MeV protons are still on their way from the Sun to the spacecraft. During later phases, in particular in connection with interplanetary shock passages (where MeV-protons are abundantly energised), protons can contaminate electrons in channels E3 and E4. During the investigated event, the SEP event had been ongoing for an extended period, exposing Mercury to sustained high fluxes of both energetic electrons and protons. However, during the Mercury flybys, the planetary magnetic field and the planet itself can shield against one species but leave the other with access to the detector, so care needs to be taken to correctly interpret variations in E3–E4 and P1–P2 channels.

To assess possible contamination during the electron and proton dropouts, we converted electron channels E3 and E4 to equivalent proton fluxes and projected them onto the proton energy spectra and conversely converted proton channel P1 to an equivalent electron flux for comparison with the electron energy spectra (Extended Data Figs. 4 and 5).

During the electron dropout (Extended Data Fig. 5; 21:47–21:50 UT) we focus on observations on sides 1 and 2 as both the electron and proton fluxes on side 0 are low. On side 1, channels E1, E2, E5 and E6 exhibit a consistent energy spectrum, allowing for a linear fit with a slope of −2.35 consistent with typical values observed during the investigated event, whereas applying electron channels E1–E6 gives a considerable flatter (harder) slope as indicated in Fig. 6b. Channels E3 and E4, as well as the converted P1 flux (blue star), lie considerably above the line fitted to channels E1, E2, E5 and E6 with their flux values increasing with energy. Such steeply rising spectra are unlikely to be physical and a more plausible explanation for the elevated E3 and E4 fluxes is a proton contamination. This interpretation is supported by the converted E3 and E4 fluxes (Extended Data Fig. 5d, purple diamonds) matching well with the proton energy spectra. Side 2 exhibits approximately similar trends as side 1. Consequently, during this interval, higher fluxes on side 1 and side 2 when compared with side 0 are probably due to proton contamination, indicating that nearly all electrons are pushed to the surface.

During the side 0 proton dropout (Extended Data Fig. 4; 21:51–21:53 UT), side 0 electron channels E1, E2 and E6 show an energy spectrum with a slope of −2.67, which matches well with the typical values observed during this event. By contrast, channels E3–E5 and the converted P1 flux (blue star) display a bump relative to the fitted line. This enhancement peaking at ~200–400 keV with PAs being close to 90° (Fig. 4f) suggests a locally trapped population as discussed in the Results. These electrons are, therefore, probably contaminating the lowest proton channels. This interpretation is further supported by the converted E3 and E4 fluxes (Extended Data Fig. 4, purple diamonds) and the P1 flux deviating considerably from the trend determined from the P2 and P3 channels. In addition, similar to very sharply rising spectra, a very steep decrease of ~1-MeV proton fluxes with increasing energy would unlikely to be physically produced in this situation. Sides 1 and 2 show in turn very similar behaviour as during the electron dropout, suggesting that protons keep contaminating electron channels E3 and E4 (On sides 1 and 2, no proton flux drop-out occurred).

In summary, the analysis indicates that during the investigated times side 0 electrons are contaminating proton channels, while on sides 1 and 2, protons contaminate electron channels.

Supplementary Figs. 2–7 also demonstrate that contamination is not an issue during other periods when both electron and proton fluxes are at high levels, we present the 2-min electron and proton spectra for the entire flyby interval. Outside the dropout periods, the electron (E1–E6) and proton (P1–P7) spectra are fitted robustly and the converted proton channel P1 and electron channels E4 and E5 show deviations from the fitted lines. On side 0, during the interval when the electron fluxes have recovered but the proton drop-out is still ongoing (~21:58–22:04), proton flux enhancements near the one-count level are probably due to electron contamination. After 22:06 UT, minor proton contamination may be present; however, the electron channels remain dominated by electrons.

### CME and flare information

Information on the CME and flare occurrences is available through various public resources. In this study, we utilized entries from the SOHO/LASCO catalogue^53^ and the STEREO SEEDS catalogue^54^ for CMEs. Flare occurrences were obtained from the Solar Orbiter STIX list^55^ and Solar Monitor (https://solarmonitor.org). The multi-spacecraft longitudinal plot in Fig. 1 was compiled using the Solar-MACH tool (https://solar-mach.github.io/).

### KTH22 model and particle motion

The semi-empirical Korth–Tsyganenko–Heyner 2022 (KTH22) model calculates the magnetic field within the Hermean magnetosphere as described in ref. ^24^. The model was originally adapted by ref. ^22^ of ref. ^56^ Earth’s magnetic field model, and later improved upon by ref. ^23^. It includes different modules to describe the internal dipole field, neutral sheet current and eastward ring current. The KTH22 model has been calibrated with MESSENGER observations, and it provides an adequate description of both the field direction and its magnitude. For the magnetic field residuals, see ref. ^24^.

The model is controlled by the DI based on MESSENGER observations^57^. It describes how disturbed the magnetosphere is, ranging from 0 (quiet) to 100 (highly disturbed). We use a DI value of 27 and a solar wind speed of 400 km s^−1^, corresponding to a weakly disturbed magnetosphere. These parameters provide the best match to the outbound magnetopause crossing, as detected by MPO/MAG 1-min data (Extended data Fig. 1). The outbound crossing is not clearly evident from the data and the field shows more variations.

The magnetic field is given either in the Mercury-Solar-Orbital (MSO) or Mercury-Solar-Magnetospheric (MSM) coordinate systems. In both cases, the *x* axis is directed to the Sun, the *z* axis points to the north and the *y* axis points in the opposite direction to the motion of Mercury about the Sun, completing the right-handed system. The difference is in the origin of the system; in MSO it is placed at the centre of the planet, whilst in MSM it is shifted northwards from the centre to consider Mercury’s dipole offset.

The magnetic field components are calculated using the KTH22 model as 1 s intervals to coincide with SIXS high resolution cadence during the flyby. However, the mirror ration and loss cone angles (Fig. 4) are calculated as at 30 s intervals due to more time-intensive field line tracing performed at each timestamp. In Fig. 4 from the start of the plot at 21:29:30 UT to 21:38:10 UT, the dot product of the magnetic field vector ($\mathbf{B}$) with the boresight viewing direction vector ($\mathbf{D}$) is negative ($\mathbf{B}\cdot \mathbf{D} < 0$) so the surface magnetic field chosen for this range is the northern hemisphere footpoint. From 21:38:20 UT to 21:52:30 UT, $\mathbf{B}\cdot \mathbf{D} > 0$ so the surface magnetic field chosen for this range is the southern hemisphere footpoint. From 21:52:40 UT to the end of the plot at 21:59:30 UT, $\mathbf{B}\cdot \mathbf{D} < 0$ so the surface magnetic field chosen for this range returns to the northern hemisphere footprint.

The mirror ratio (${\mathcal{R}}$) is defined as the ratio of the magnetic field magnitude at the spacecraft location to the maximum magnetic field magnitude encountered along the field line from the spacecraft towards the northern (${{\mathcal{R}}}_{{\mathcal{N}}}$) and southern hemispheres (${{\mathscr{ \mathcal R }}}_{{\mathscr{ \mathcal N }}}$). These are considered separately because the magnetic field magnitude in the southern hemisphere can be up to three times weaker than in the northern hemisphere, for example, ref. ^41^. As energetic electrons bounce extremely rapidly along magnetic field lines, they can precipitate into either hemisphere. When mirror ration is below unity electrons with all PA could precipitate.

The loss cone (LC) critical angle is calculated for each point using

1 $${\alpha }_{LC}=\arcsin \frac{1}{\sqrt{{\mathcal{R}}}},$$

separately for the southern and northern hemisphere mirror ratios (${\mathcal{R}}_{\mathcal{N}}$ and ${\mathcal{R}}_{\mathcal{S}}$). The mirror ratios and loss cone angles are shown in Fig. 4.

### Toffoletto–Hill model

We used the PLANETOCOSMICS^58,59^ Geant4 application to simulate the propagation of charged particles in Mercury’s magnetosphere by solving the equation of motion in a given planetary magnetic field. Such computations allow us to determine if a particle can reach BepiColombo’s location in a specific direction from the magnetopause (that is, allowed trajectory) or not (forbidden trajectory) at a selected time. Note that trajectories that would normally be allowed in a pure magnetic field become forbidden due to the presence of the planet. Trajectories are computed backwards from the spacecraft position to the magnetopause. As for the planetary magnetic field, we used the modified version of the semi-analytical Toffoletto and Hill magnetospheric model (TH93 refs. ^28,60^), scaled and adapted to Mercury^61^.

In the TH93 code, the inner field, given by the Voigt model^62^, is completely shielded by the magnetopause, which is approximated by a semi-sphere on the dayside (with its centre shifted antisunwards, with respect to the planet, depending on the solar wind dynamic pressure) and by a cylinder on the tail side. The magnetic reconnection with the interplanetary magnetic field (IMF) is obtained by providing the distribution of the normal component along the dayside magnetopause, which depends upon the IMF orientation. The ring-current contribution was removed from the original TH93 code; the interconnection coefficients of the dipole and IMF fields were set equal to 0.1 and 0.9, respectively; the tail field strength and stretching factors were set equal to 10 and 0.1, respectively; while the coefficients controlling the shape and location of the X-line in the tail, L(0) and L(mp), were set to 4 and 6, respectively; the plasma sheet thickness was set equal to 0.1–0.4, according to ref. ^63^. To calculate the radius of the (semi-spherical) dayside magnetopause as a function of the solar wind conditions, the empirical relation of refs. ^64,65^, was adopted

2 $$\begin{array}{ll}r \, = \, {r}_{0}{\left(\frac{2}{1+\cos \theta }\right)}^{\alpha }\\ \alpha \, = \, \left(0.58-0.007{B}_{z}\right)\left(1+0.024\mathrm{log}{P}_{\mathrm{dyn}}\right),\end{array}$$

where both *r*_0_ and *α* depend on IMF *B*_*z*_ component (nT) and solar wind dynamic pressure *P*_dyn_ (nPa). In the present analysis, we have adopted a dipole magnetic moment of ${R}_{M}^{3}$, and a northward dipole shift of 0.2 *R*_*M*_ (ref. ^40^). To calculate *P*_dyn_, we assumed nominal values for the solar wind density and velocity. The MAG instrument^9^ on board BepiColombo measured the input IMF components values. In this configuration, we computed the particle trajectories for 55, 47 and 51 energies in the three channels corresponding to the P1 + P2, P5 + P6 and P7 + P8, respectively (assuming a flat spectrum in each channel). We modelled the protons arrival to the spacecraft’s position from all the directions having zenith angle in the range [0, 180] degree and azimuth [0, 360] at steps of 1°. Finally, we obtained the percentage of allowed trajectories for the considered directions and channels. We performed such simulations for the following times: 21:43, 21:49, 21:53, 21:55, 22:01 and 22:07 (all UTC).

## Supplementary information

Supplementary Information Supplementary Figs. 1–7.

## Data Availability

The SIXS-P measurements, KTH22 modelling results, including the calculated mirror ratios, loss cone widths and electron PAs, as well as KTH modelling results are made available via Zenodo at https://zenodo.org/uploads/20089141 (ref. ^66^).

## Code availability

The KTH22 model code is available via GitHub at https://github.com/Krissy37/KTH-V10.
