# Supplementary material for: Planetary shielding and surface precipitation of solar energetic particles during BepiColombo’s close Mercury flyby
Source: Nat Astron. 2026 Sep 8;10(10):1478–88. doi: 10.1038/s41550-026-02914-6 (PMC13672065; doi:10.1038/s41550-026-02914-6)
Supplement: Supplementary file 1 [file 41550_2026_2914_MOESM1_ESM.pdf]

nature astronomy

Supplementary information https://doi.org/10.1038/s41550-026-02914-6

Planetary shielding and surface precipitation of solar energetic particles during BepiColombo's close Mercury flyby

In the format provided by the authors and unedited

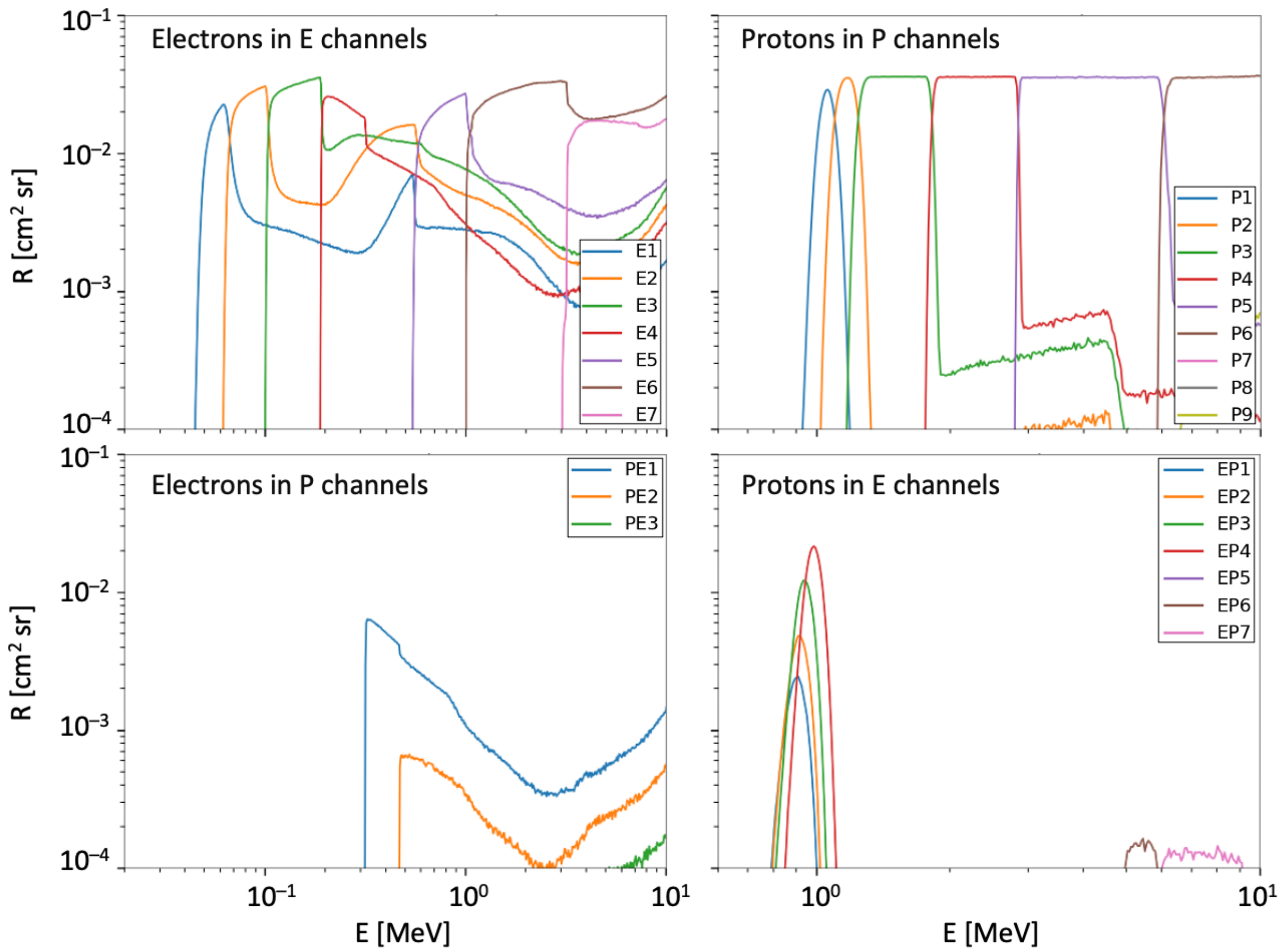

Supplementary Fig. 1 SIXS-P response functions, Side 0. SIXS-P Side 0 response functions for electron (E) and proton (P) channels. Top row) nominal particle species; Bottom row) contaminating species.

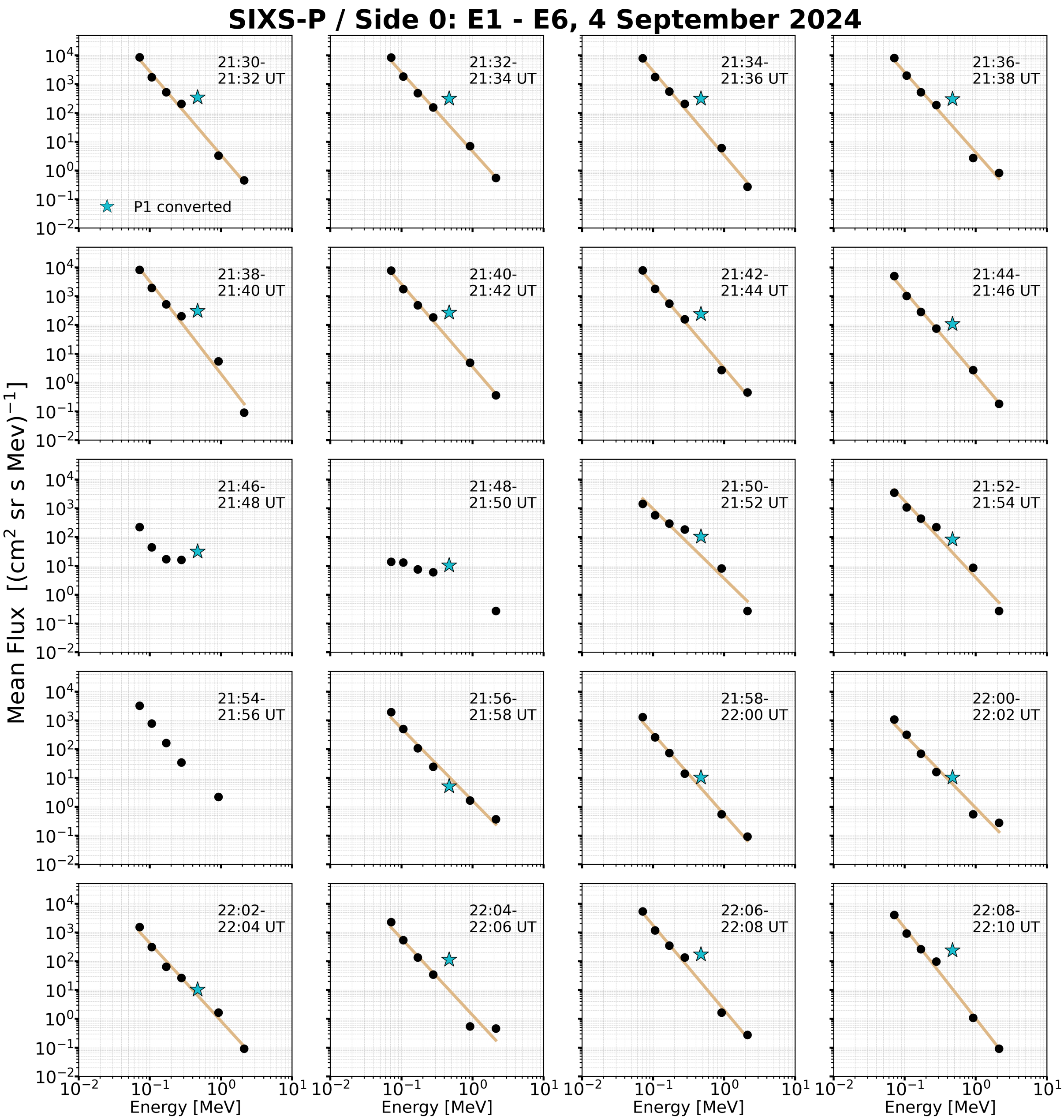

Supplementary Fig. 2 Electron energy spectra (full interval), Side 0. The electron spectra and fit for channels E1- E6. The proton channel P1 converted to electron channel is shown as a blue star. The spectra are compiled for 2 minutes intervals.

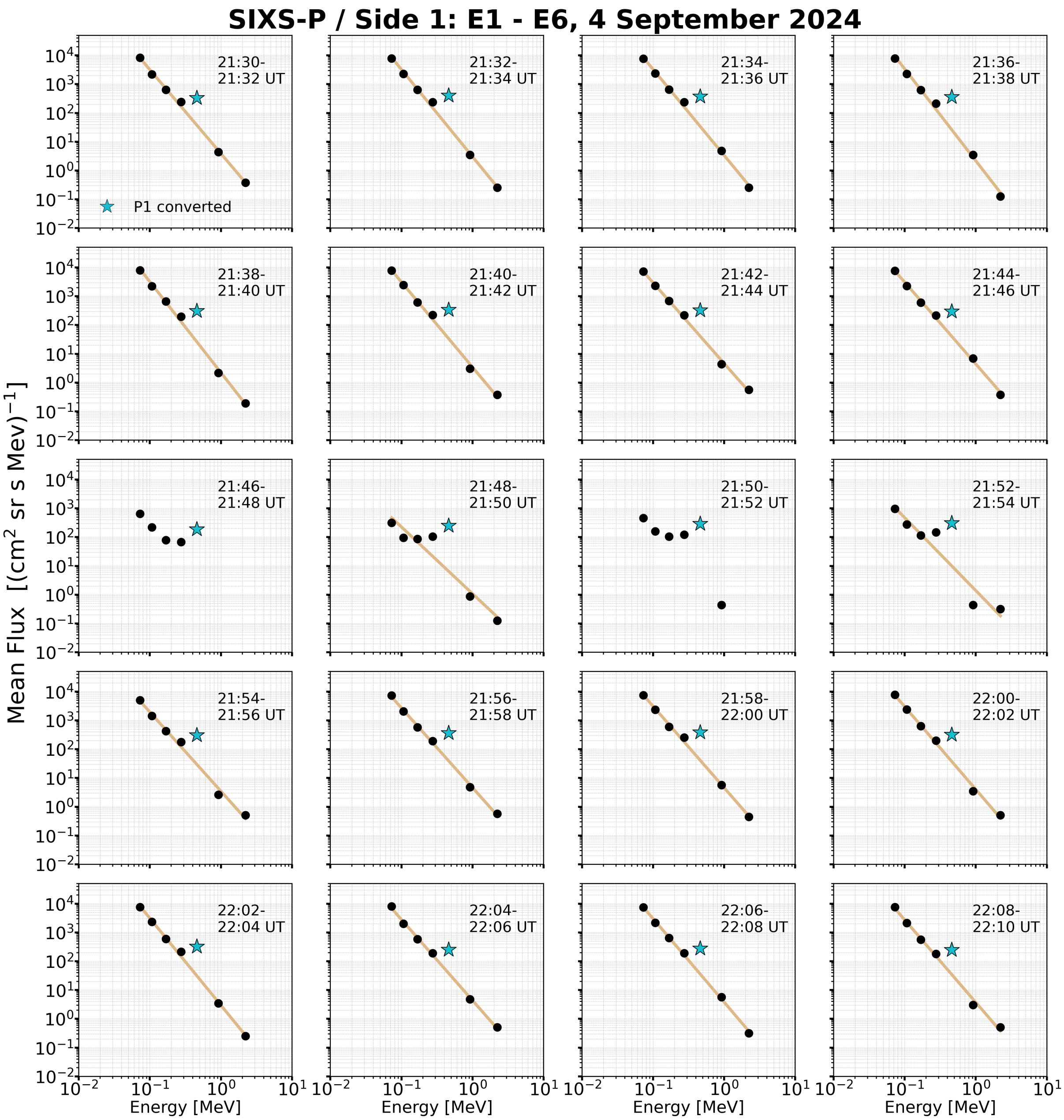

Supplementary Fig. 3 Electron energy spectra (full interval), Side 1. Same as Supplementary Fig. 2 but for the Side 1.

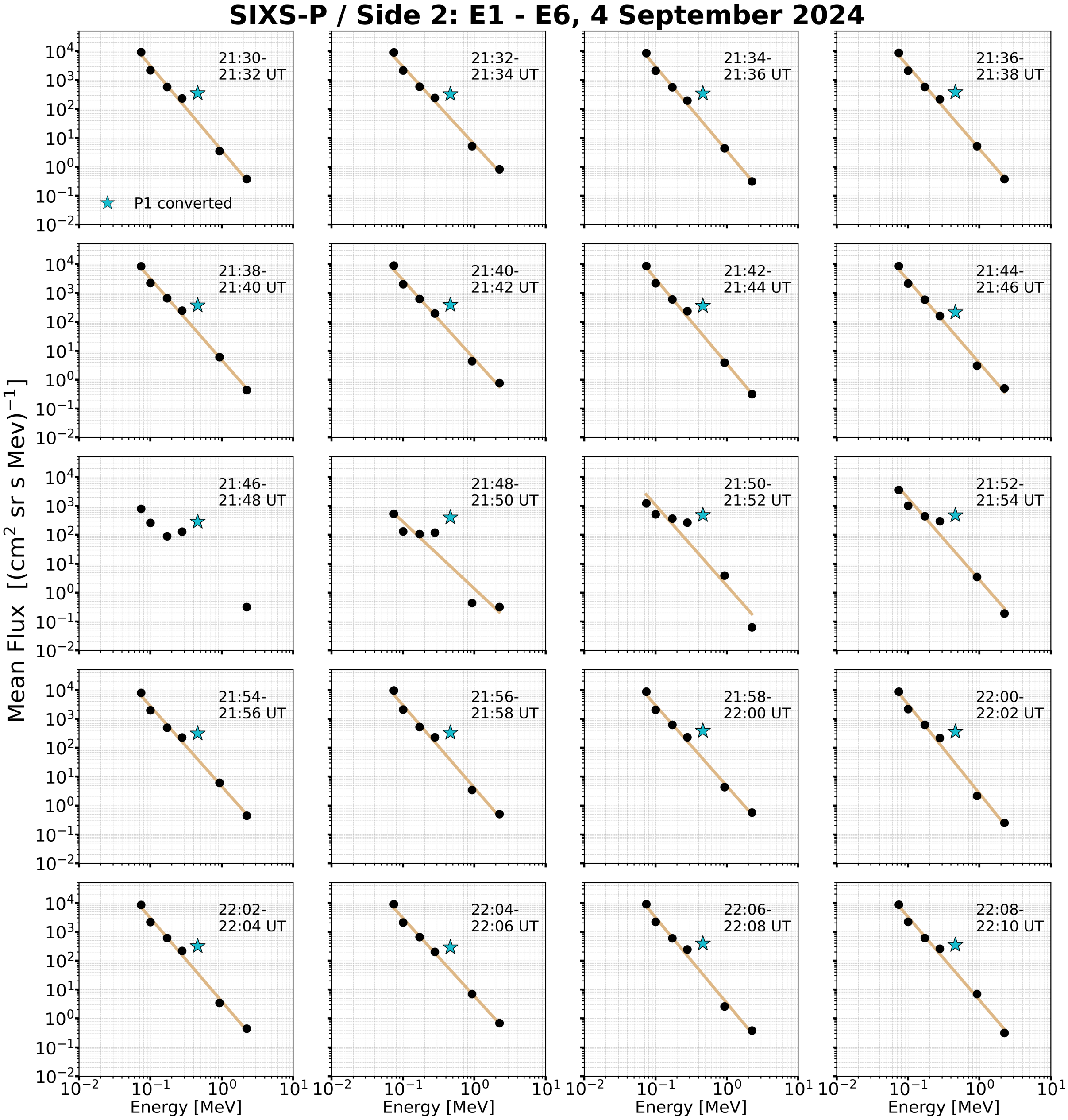

Supplementary Fig. 4 Electron energy spectra (full interval), Side 2. Same as Supplementary Fig. 2 but for the Side 2.

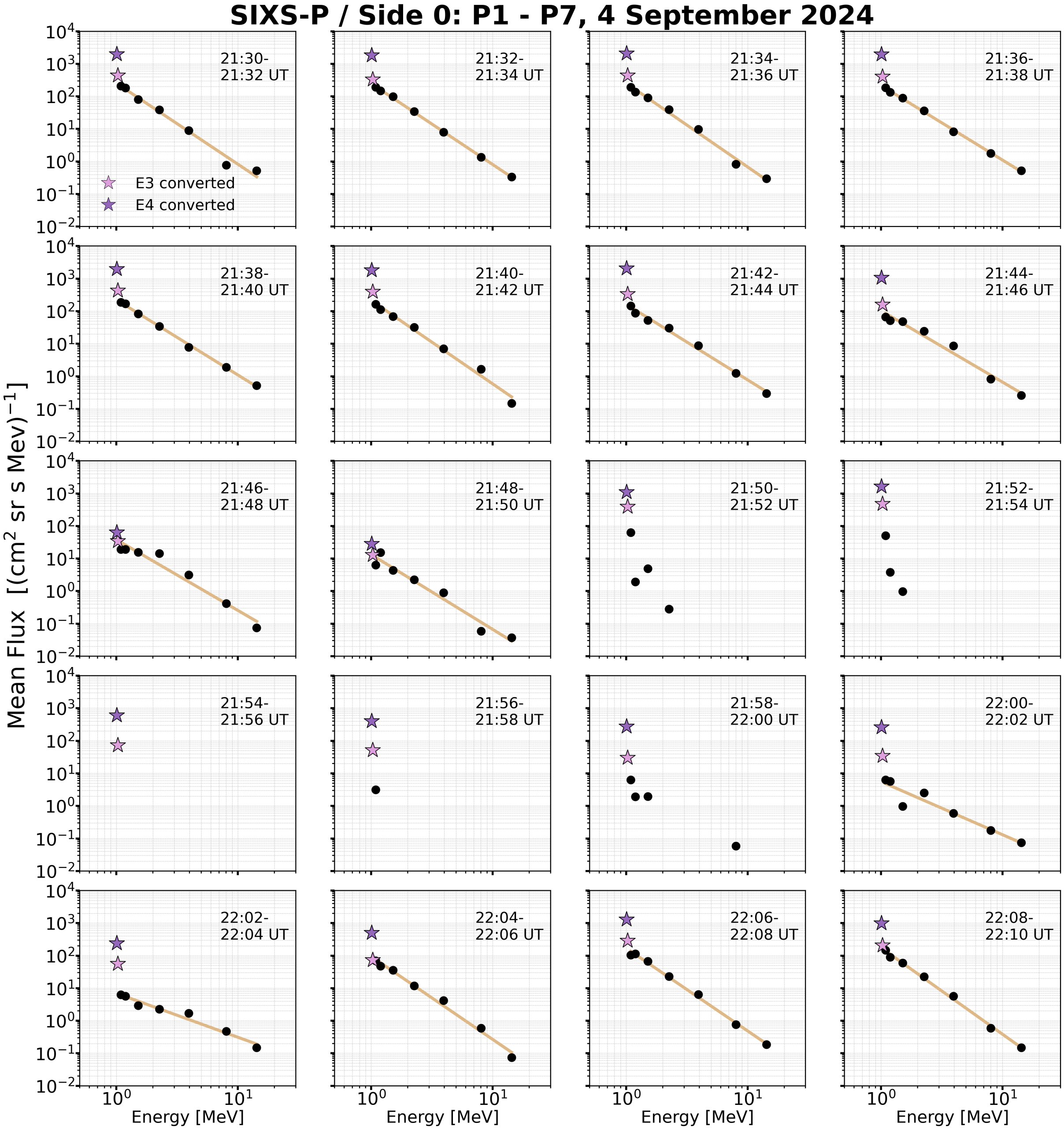

Supplementary Fig. 5 Proton energy Spectra (full interval), Side 0. The proton spectra and fits for channels P1-P7. The electron channels E3 and E4 converted to proton channels are shown as purple stars. The spectra are compiled for 2 minutes interval from 21:30 to 22:10 UT.

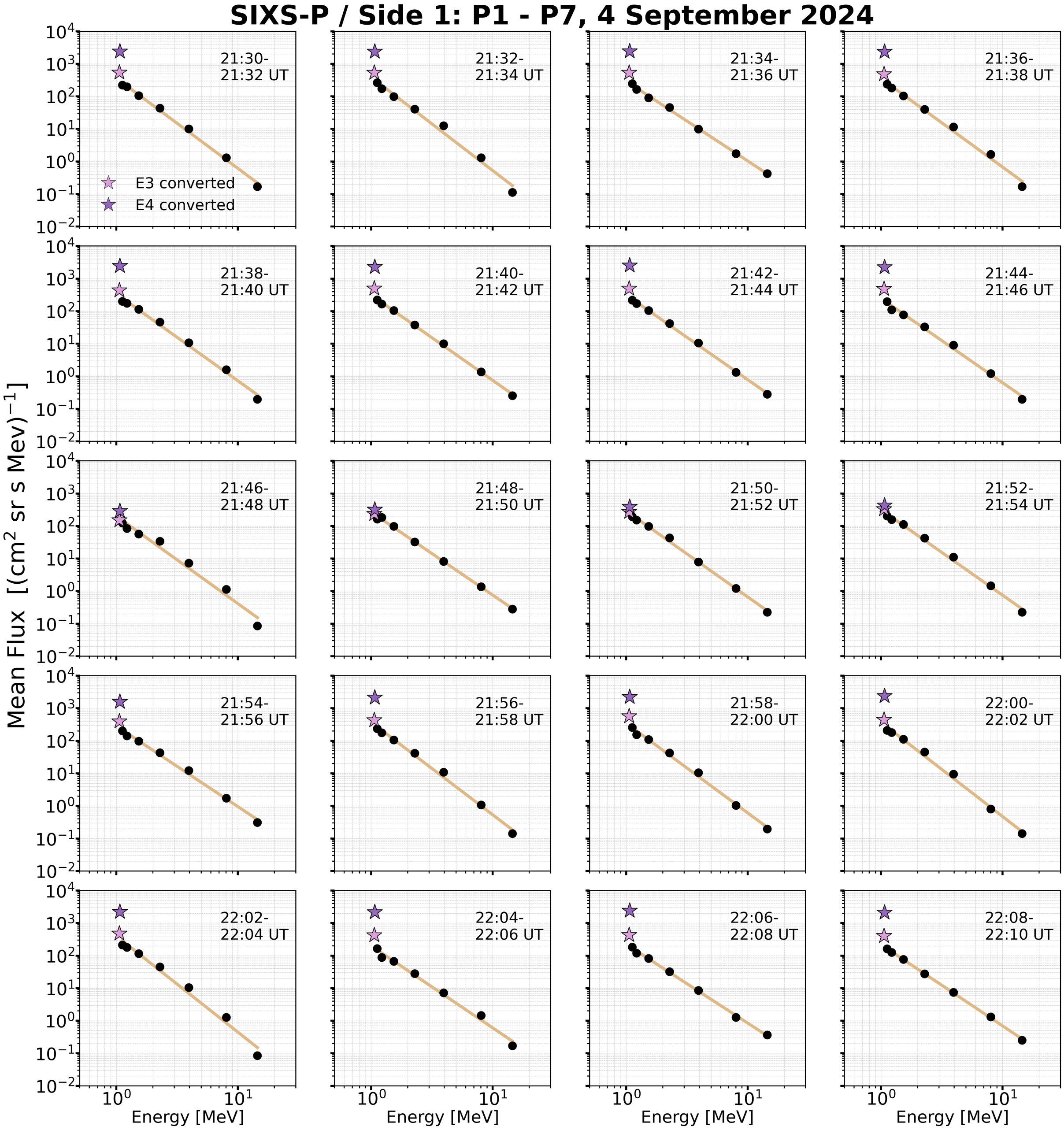

Supplementary Fig. 6 Proton energy Spectra (full interval), Side 1. Same as Supplementary Fig. 5 but for the Side 1.

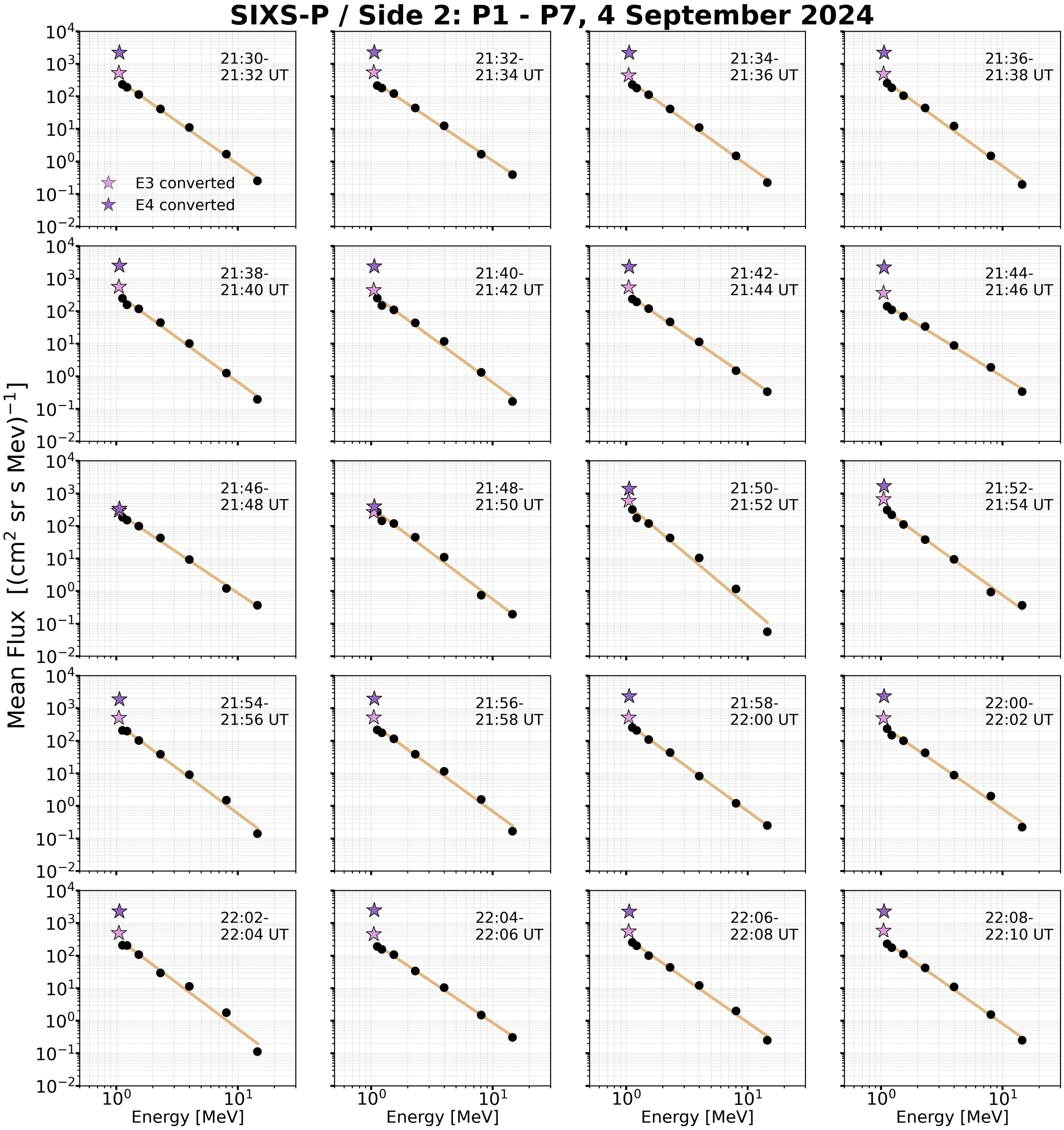

Supplementary Fig. 7 Proton energy Spectra (full interval), Side 2. Same as Supplementary Fig. 5 but for the Side 2.
